# Exploring immune-endothelial interactions in neuroinflammation and vascular dysfunction

**DOI:** 10.3389/fnmol.2026.1978704

**Published:** 2026-10-14

**Authors:** Zitian Wang, Zezheng Kang, Lei Wu, Jinglin Hu, Xinhua Chen, Tuoxian Tang

**Affiliations:** 1 Changchun University of Chinese Medicine, Changchun, China; 2 Affiliated Hospital of Changchun University of Chinese Medicine, Changchun, China; 3 Department of Biology, University of Pennsylvania, Philadelphia, PA, United States

**Keywords:** blood-brain barrier, cerebrovascular endothelial dysfunction, immune-endothelial-neural axis, neurodegenerative diseases, neuroinflammation, neurovascular unit, traditional Chinese medicine

## Abstract

Neuroinflammation and cerebrovascular endothelial dysfunction are traditionally studied as parallel processes confined to individual disease categories. Yet a growing body of evidence indicates that they operate as components of a tightly coupled, self-amplifying circuit: the immune-endothelial-neural axis. Within this axis, the peripheral immune system, cerebrovascular endothelium, and neural networks continuously modulate one another across disease contexts. This Review summarizes current knowledge on the bidirectional immune-endothelial interactions that underlie blood-brain barrier disruption and neuroinflammation across cerebrovascular disorders, neuroimmune diseases, and neurodegenerative conditions. A systems-level comparison across disease categories identifies shared pathological hallmarks alongside disease-specific signatures of tempo, vascular scale, and regional vulnerability. Emerging therapeutic strategies are considered along the three limbs of the axis: immunomodulation, endothelial protection and barrier stabilization, and neural recovery, including traditional Chinese medicine as a multitarget approach. By framing neurological disorders through the immune-endothelial-neural axis, this Review proposes a unifying framework that bridges disease boundaries and identifies common mechanistic targets for future translational research. The framework is offered as a testable proposition in which the relative contribution of each limb differs between disorders.

## 1 Introduction

Neuroinflammation refers to inflammatory processes within the central nervous system (CNS), characterized by activation of resident immune cells (microglia, astrocytes, and CNS-associated macrophages) and often accompanied by peripheral immune cell infiltration (Prinz and Priller, 2014; DiSabato et al., 2016). Under physiological conditions, tightly regulated neuroinflammation contributes to tissue homeostasis, synaptic plasticity, and pathogen clearance (Parkhurst et al., 2013; Tremblay et al., 2011). When inflammatory stimuli persist, or regulatory mechanisms fail, however, glial cells transition from transient

surveillance to chronic activation. They accumulate excessive pro-inflammatory mediators that shift immune-glia-neuron signaling from repair to injury. This shift ultimately drives synaptic dysfunction, axonal damage, and neuronal apoptosis (DiSabato et al., 2016; Liddelow et al., 2017; Hong et al., 2016).

Cerebrovascular endothelial dysfunction describes the phenotypic conversion of brain microvascular endothelial cells from a quiescent, protective state (maintaining anti-inflammatory, antithrombotic, and low-permeability properties) to a pathological state marked by pro-inflammatory, pro-coagulant, and hyperpermeable features (Galea, 2021; Sweeney et al., 2018). Brain endothelial cells constitute the structural core of the blood-brain barrier (BBB). Tight junctions (claudin-5, occludin, ZO-1), adherens junctions, and selective transport systems collectively seal the paracellular space while controlling transcellular trafficking of nutrients and waste (Abbott et al., 2010; Hawkins and Davis, 2005). They further preserve microvascular homeostasis by synthesizing vasodilators such as nitric oxide (NO) and prostacyclin (PGI_2_), which inhibit leukocyte and platelet adhesion. Chronic exposure to hypertension, hyperglycemia, dyslipidemia, and systemic inflammation is associated with a shift in this equilibrium. This shift is mediated by declining NO bioavailability, a surge in reactive oxygen species (ROS), upregulation of adhesion molecules (ICAM-1, VCAM-1, E/P-selectins), and deterioration of junctional integrity. These changes impair vasomotor regulation, promote microthrombosis, and compromise BBB integrity (Sweeney et al., 2018; Taddei et al., 2008).

Clinical and experimental data converge on a recurring observation across cerebrovascular, neuroimmune, and neurodegenerative disorders. Neuroinflammation and endothelial dysfunction do not operate independently but instead reinforce one another in a self-amplifying loop (Wang et al., 2025; Sweeney et al., 2019). Endothelial activation and BBB disruption open a gateway for peripheral immune cells and inflammatory mediators to enter the brain, lowering the threshold for neuroinflammation. Sustained neuroinflammation, in turn, exacerbates oxidative stress, vascular remodeling, and microcirculatory failure (Candelario-Jalil et al., 2022). This bidirectional coupling recurs across a broad disease spectrum. In cerebrovascular disorders, including ischemic stroke, intracerebral hemorrhage (ICH), and cerebral small vessel disease (CSVD), acute ischemia-reperfusion injury, blood degradation products, or chronic hemodynamic stress. These converge on endothelial activation, BBB breakdown, and immune-mediated secondary damage. These processes drive outcomes from hemorrhagic transformation to progressive white matter injury (Jurcau and Simion, 2021). In neuroimmune diseases such as multiple sclerosis (MS) and neuromyelitis optica spectrum disorder (NMOSD), pathogenic T and B lymphocytes traverse the BBB via adhesion molecules and chemokine gradients. This establishes cycles of immune cell infiltration, endothelial injury, and self-perpetuating neuroinflammation (Engelhardt and Ransohoff, 2012; Sallusto et al., 2012). Even in neurodegenerative diseases long viewed as primarily proteinopathic, including Alzheimer's disease (AD) and Parkinson's disease (PD), chronic systemic inflammation, endothelial dysfunction, and subtle BBB leakage increasingly appear as integral components of pathogenesis. They also interact with amyloid-β (Aβ) and α-synuclein aggregation to accelerate neurodegeneration (Sweeney et al., 2018; Tansey et al., 2022). The initiating event differs between disorders. In ischemic stroke it is ischemia-reperfusion injury, and in intracerebral hemorrhage it is the release of blood degradation products and iron. In multiple sclerosis and neuromyelitis optica spectrum disorder it is antigen-specific immune attack, whereas in cerebral small vessel disease and vascular cognitive impairment it is chronic haemodynamic and metabolic burden. In Alzheimer's disease and Parkinson's disease, it is protein aggregation together with vascular aging. Depending on the disorder and stage, the endothelium may therefore act as the site of initiation, an amplifier of inflammation, or a downstream consequence of it. The axis is best read as a bidirectional circuit rather than as a fixed sequence of events.

Taken together, these observations motivate a conceptual shift (Figure 1). Rather than treating neuroinflammation and vascular dysfunction as parallel processes within individual diseases, it may be more productive to view them as components of a tightly coupled “immune-endothelial-neural axis.” This axis is a dynamic, bidirectional system in which the peripheral immune system, cerebrovascular endothelium, and neural networks interact at multiple levels to determine disease initiation, progression, and outcome (Iadecola, 2017; Kobeissy and Salzet, 2026). This framework unifies seemingly heterogeneous conditions across cerebrovascular, neuroimmune, and neurodegenerative categories. It also suggests that common pathological pathways (and therefore common therapeutic targets) may exist across traditional diagnostic boundaries (Zlokovic, 2011; Lapides and McDonald, 2020). This review examines the structural and functional basis of this axis (Section 1.1), maps its manifestations across disease spectrums (Sections 2 and 3), and explores its translational implications for biomarker development and therapeutic intervention (Sections 4 and 5).

**Figure 1:**
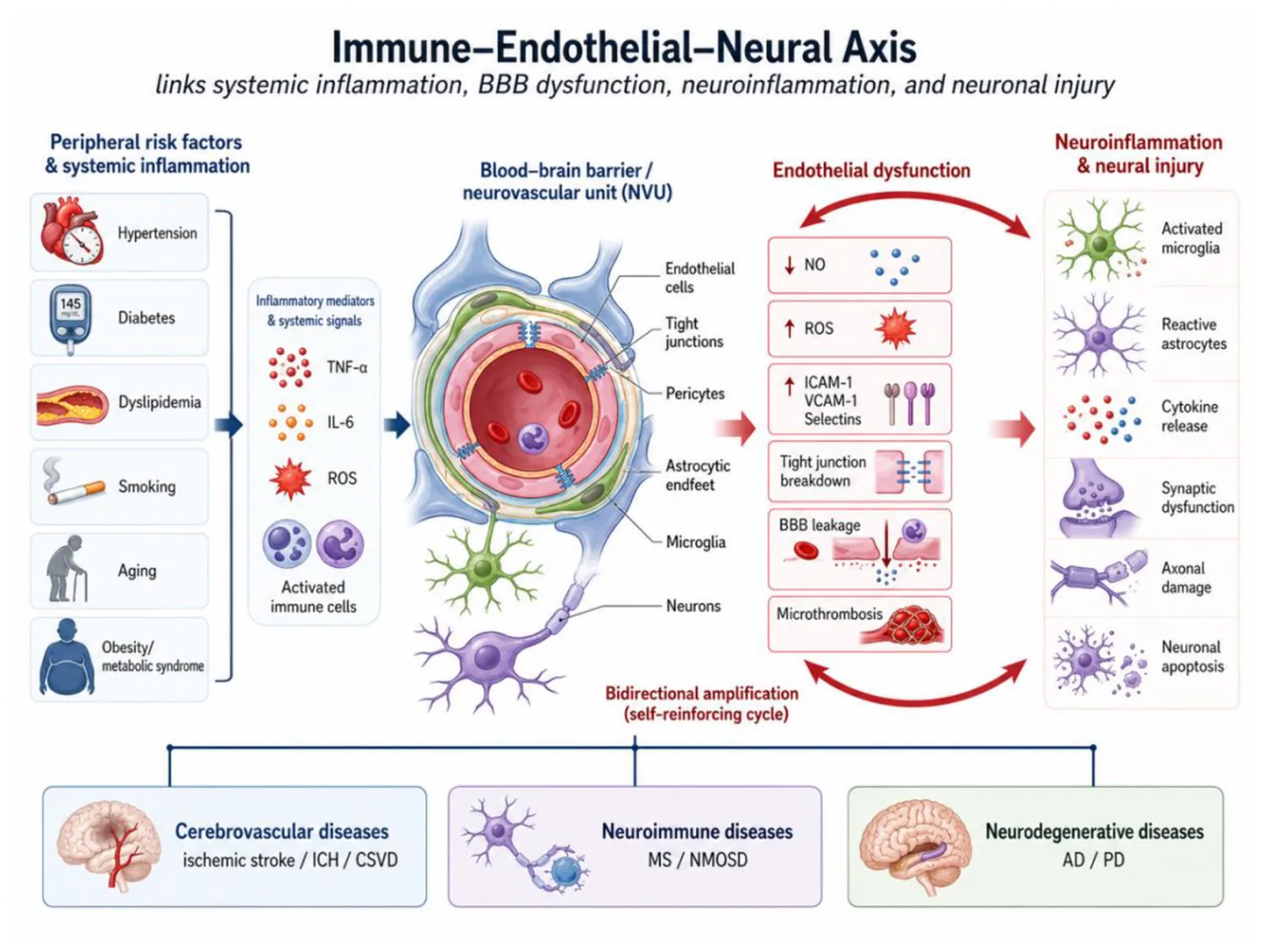
The immune-endothelial-neural axis: a unified pathophysiological framework. Left panel: Traditional vascular risk factors (hypertension, diabetes, dyslipidemia, smoking, aging, and metabolic syndrome) are associated with chronic systemic inflammation characterized by elevated TNF-α, IL-6, reactive oxygen species (ROS), and activated peripheral immune cells. Center panel: These inflammatory mediators act on the blood-brain barrier (BBB), whose neurovascular unit (NVU) comprises endothelial cells sealed by tight junctions, pericytes embedded in the basement membrane, astrocytic endfeet, microglia, and neurons. Upper right panel: Endothelial cells undergo progressive phenotypic transition, including decreased NO bioavailability, increased ROS production, upregulation of adhesion molecules (ICAM-1, VCAM-1, selectins), tight junction breakdown, BBB leakage, and microthrombus formation. Far right panel: BBB disruption permits immune cell infiltration and blood-derived mediators to reach the CNS parenchyma, triggering microglial activation, astrogliosis, cytokine release, synaptic dysfunction, axonal damage, and neuronal apoptosis. Red bidirectional arrows: A self-reinforcing amplification loop in which neuroinflammation further damages the cerebrovascular endothelium, creating sustained pathological cycling. Bottom: Three disease categories—cerebrovascular diseases (ischemic stroke, ICH, CSVD), neuroimmune diseases (MS, NMOSD), and neurodegenerative diseases (AD, PD)—share this common axis, differing in tempo, vascular scale, and regional distribution.

### 1.1 The neurovascular unit and blood-brain barrier: structural basis of immune-endothelial interactions

The neurovascular unit (NVU) is a functionally integrated system. Brain microvascular endothelial cells, pericytes, astrocytic endfeet, microglia, neurons, and the extracellular matrix operate as a coordinated whole rather than a mere assemblage of adjacent cell types (Muoio et al., 2014; Wälchli et al., 2024). Within this arrangement, endothelial cells form the luminal lining of cerebral capillaries; pericytes, embedded in the shared basement membrane, regulate vascular tone, and endothelial stability; astrocytic endfeet ensheath the abluminal surface, coupling neural activity to vascular responses while mediating water and ion exchange; and resident microglia continuously surveil the perivascular microenvironment (Ayloo et al., 2022; Montagne et al., 2020). Neurons complete the circuit by signaling local metabolic demand through neurotransmitters and neurotrophic factors (Yabluchanskiy et al., 2021; Attwell et al., 2010; Longden et al., 2017).

Built on this structural foundation, the BBB transforms the NVU into a selective interface (He et al., 2026; Chen T. et al., 2024). Cerebral endothelial cells differ from their peripheral counterparts in several critical aspects. They exhibit minimal fenestration and transcytosis, and are sealed by highly developed tight and adherens junctions that collectively block passive paracellular diffusion (Abbott et al., 2010; Hawkins and Davis, 2005; Sweeney et al., 2019). The basement membrane, co-secreted by endothelial cells and pericytes, adds a second physical layer and anchors adhesion molecules (Thomsen et al., 2017). Astrocytic endfeet, expressing aquaporin-4 (AQP4), potassium channels, and glutamate transporters, form a third functional layer that regulates water balance, synaptic waste clearance, and vascular signaling (Liao et al., 2024; Krings et al., 2025). Together, these three concentric layers (endothelium, basement membrane, and glia limitans) create a multistage barrier that restricts the entry of most circulating macromolecules and cells into the CNS parenchyma.

The same endothelial cells that maintain this physical barrier simultaneously act as the primary signaling interface between the peripheral immune system and the brain (Sweeney et al., 2019; He et al., 2026). Their luminal surface displays adhesion molecules, chemokines, and immune receptors that govern leukocyte recognition, rolling, adhesion, and transendothelial migration (Marchetti and Engelhardt, 2020; Ley et al., 2007). Their abluminal side releases paracrine signals that modulate pericyte contractility, astrocytic endfoot activity, and microglial surveillance. Under steady-state conditions, this dual identity is favorable for protection: low adhesion molecule expression, minimal cytokine secretion, and intact junctional complexes keep immune cell trafficking to a trickle (Louveau et al., 2015). During inflammation, however, endothelial cells rapidly shift toward an activated phenotype, upregulating adhesion molecules, secreting chemokines, and remodeling junctional complexes. These changes convert the BBB from a selective filter into a permissive gateway (Sweeney et al., 2019; He et al., 2026). It is this phenotypic plasticity that positions brain endothelial cells at the pivot of the immune-endothelial-neural axis. They receive inputs from the circulation, the immune system, and the neural parenchyma. They translate these signals into changes in barrier permeability, vascular tone, and inflammatory tone that directly shape the brain's response to injury (Widlansky et al., 2003; Erickson and Banks, 2018).

### 1.2 From traditional vascular risk factors to the “immune-endothelial-neural axis”

Stroke, CSVD, and VCID have long been classified as vascular conditions, with their risk profiles dominated by hypertension, diabetes, dyslipidemia, smoking, and aging (Kapasi and Schneider, 2016; Sachdev et al., 2026). Previous studies suggested a straightforward pathogenesis for these diseases. Chronic hemodynamic stress and vascular injury accumulate over years, progressively degrading the cerebral microvasculature until a threshold of structural damage is crossed and clinical disease emerges (Pantoni, 2010; Luo and Qiao, 2026; Wardlaw et al., 2019). However, this framing captures only half the picture.

A substantial body of work now indicates that traditional risk factors do not act exclusively through mechanical stress on vessel walls (Gasmi et al., 2021). Obesity, type 2 diabetes, and metabolic syndrome are associated with a state of chronic low-grade systemic inflammation marked by elevated TNF-α, IL-6, and activated innate immune cells (Hotamisligil, 2017; Rohm et al., 2022). Hypertension reshapes circulating immune cell populations toward pro-inflammatory phenotypes and increases endothelial oxidative stress independently of blood pressure elevation *per se* (Nguyen et al., 2024; Griendling et al., 2021). Dyslipidemia and atherosclerosis are chronic inflammatory conditions driven by monocyte infiltration and sustained vascular inflammation (Chen X. et al., 2024; Moore and Tabas, 2011). Smoking maintains peripheral neutrophils and monocytes in a pre-activated state, and aging brings the cumulative phenomenon of “inflammaging”: a gradual rise in systemic inflammation accompanied by declining immune regulatory capacity (Derella et al., 2021; Li et al., 2023). Despite their clinical diversity, these risk factors converge on a shared immunological endpoint: chronic systemic inflammation that chronically primes cerebral endothelial cells toward activation (Galea, 2021; Li et al., 2023).

The consequence is not a one-way cascade but a self-reinforcing circuit. Peripheral inflammatory signals act on cerebral endothelial cells through circulating cytokines, metabolic disturbances, and altered hemodynamics. This shifts them from a protective to an activated phenotype, upregulating adhesion molecules, remodeling junctional complexes, and increasing permeability (Galea, 2021; Chmielarz et al., 2024). The resulting BBB disruption permits immune cells and inflammatory mediators to enter the CNS, initiating or amplifying neuroinflammation (Linnerbauer et al., 2020; Wang et al., 2026). Critically, neuroinflammation does not stop at the parenchyma: glial activation releases soluble mediators that feed back to the vasculature, further impairing endothelial function and BBB integrity (Abbott et al., 2006). In this model, traditional vascular risk factors can be understood as conditions associated with progressive priming of the immune-endothelial-neural axis. This is a bidirectional system in which the peripheral immune system, cerebrovascular endothelium, and neural networks continuously modulate one another (Galea, 2021). Acute events such as infection, ischemia, or hemorrhage act as triggers that push this pre-tensioned system beyond its compensatory capacity, producing overt disease (Emsley and Hopkins, 2008).

Several frameworks have addressed components of this problem in isolation. The neurovascular unit (NVU) supplies the structural substrate, defining which cell types are coupled and through which junctions and extracellular matrix. It does not, however, explicitly incorporate systemic immune dynamics (Iadecola, 2017). Neuroimmune signaling at the blood-brain barrier describes the local cellular and molecular mechanisms of immune-endothelial interaction, including adhesion, chemokine gradients, and junctional remodeling. Yet it treats the systemic immune state largely as an external input. Models of systemic inflammation in neurodegeneration, such as inflammaging, describe how peripheral cytokines reach the brain but stop short of specifying the endothelial translation step (Li et al., 2023). Peripheral-to-central signaling through the gut-brain axis and its microbial metabolites is also relevant, although its connection to cerebrovascular endothelial biology remains indirect (You et al., 2024). The immune-endothelial-neural axis is not intended to replace these frameworks but to connect them, and it is proposed as an integrative framework rather than as a newly discovered mechanism. Its specific contribution is to treat the three compartments as a single coupled circuit with defined directionality and gain. It also formalizes two elements that existing frameworks do not: first, the cerebrovascular endothelium as the obligate bidirectional translator between systemic immune state and neural parenchyma; and second, systemic immune status as the gain-setting parameter of the circuit rather than an external input. The framework is therefore judged by the predictions it generates, which are set out in Table 1.

**Table 1:** Testable predictions of the immune-endothelial-neural axis.

Prediction	Rationale	Test design	Falsified if
Endothelial priming predicts conversion after a second hit	If the axis behaves as a pre-tensioned circuit whose gain is set by systemic immune status, a measurable primed state should precede overt disease	Prospective cohort in cerebral small vessel disease or vascular cognitive impairment: baseline endothelial markers (sICAM-1, sVCAM-1, Ang-2), cerebrovascular reactivity and white matter hyperintensity burden related to subsequent cognitive conversion or new lesions	Priming markers show no predictive value after adjustment for conventional vascular risk factors
Interventions on different limbs interact, so that outcome depends on combination and sequence	If the three limbs are coupled within one circuit, modifying one limb should change the effect of modifying another	Factorial preclinical designs in middle cerebral artery occlusion and experimental autoimmune encephalomyelitis, in which an endothelial-protective agent and an immunomodulatory agent are combined in both orders and compared with either agent alone	The combination is no more effective than either agent alone and the order of administration is irrelevant
Endothelial-immune interface molecules recur across clinically distinct disorders	If a common circuit operates across disorders, the candidate target set should also be shared	Cross-disease single-cell and spatial profiling of human brain tissue and blood, testing whether interface molecules such as the ICAM-1/VCAM-1 axis, the CX3CL1-CX3CR1 axis, and the Ang-2/Tie2 axis are recurrently enriched in at least three of the disease categories discussed here	The recurrent target sets of different disorders show no significant overlap

Each prediction is specific to the immune-endothelial-neural axis, and each is stated together with the design that would test it and the observation that would falsify it. The predictions are offered as hypotheses to be tested rather than as established findings.

Viewing neurological disorders through this axis does not merely reclassify existing knowledge; it reframes the pathogenic question. Instead of asking what vascular injury has occurred, one can ask how the immune-endothelial-neural axis has been tipped out of balance, and where along its three limbs intervention might restore equilibrium (Sweeney et al., 2019). The following sections examine each limb in turn: the endothelial phenotypes that bridge vascular risk and neuroinflammation (Section 2), the cellular and molecular mechanisms of immune-endothelial crosstalk (Section 3), the shared and disease-specific features revealed by a systems perspective (Section 4), and the diagnostic and therapeutic opportunities this framework creates (Section 5).

### 1.3 Literature search and selection

This review is based on a narrative synthesis of the literature rather than on a systematic review; no formal systematic-review protocol was registered, and no quantitative pooling was performed. References were identified by searching PubMed and Web of Science. Search terms for each of the three compartments of the axis were combined with terms for each disease category considered here. For the endothelial compartment, terms included blood-brain barrier, brain endothelial cell, tight junction, adherens junction, glycocalyx and transendothelial migration; for the immune compartment, neutrophil, monocyte, macrophage, microglia, T cell, regulatory T cell, complement, cytokine and chemokine; and for the neural compartment, neuron, astrocyte, synaptic, and neurovascular coupling. These were combined with terms for ischemic stroke, intracerebral hemorrhage, cerebral small vessel disease, vascular cognitive impairment, multiple sclerosis, neuromyelitis optica spectrum disorder, Alzheimer's disease and Parkinson's disease. The search covered literature published up to June 2026 and was restricted to peer-reviewed studies in English. Priority was given to human studies and, for mechanistic questions, to studies in mammalian models. Conference abstracts, non-peer-reviewed material, and studies without extractable data on the endothelial or immune compartment were excluded. Where the available evidence was restricted to a single model system, a single cohort, or a single imaging modality, this is stated in the text. The cross-disease comparisons presented in Sections 4 and 5 are therefore a narrative synthesis and a proposed working model rather than the output of a systematic quantitative analysis.

## 2 Endothelial dysfunction as a driver of neuroinflammation and CNS vascular disease

### 2.1 From protective physiology to pathological phenotype

Under steady-state conditions, cerebrovascular endothelial cells maintain three overlapping homeostatic functions that collectively safeguard neural tissue. First, they regulate cerebral blood flow (CBF) by sensing shear stress, blood pressure, and local metabolic signals (pH, CO_2_, lactate, adenosine) and translating these into vasoactive responses. This is achieved chiefly through the balanced release of NO and PGI_2_ as vasodilators against endothelin-1 as a vasoconstrictor, thereby matching perfusion to neuronal demand through both cerebral autoregulation and neurovascular coupling (Longden et al., 2017; Xu et al., 2018). Second, they sustain a low-thrombotic, low-inflammatory microvascular environment. A glycocalyx rich in glycosaminoglycans repels inappropriate platelet and leukocyte adhesion, and surface thrombomodulin and tissue-type plasminogen activator (tPA) maintain the coagulation-fibrinolysis equilibrium. Minimal expression of adhesion molecules (ICAM-1, VCAM-1, E-/P-selectins) and MHC class II keeps the cerebral vascular wall largely invisible to circulating immune cells (Ando et al., 2018). Third, as the structural core of the BBB, they seal the paracellular space through tight junctions and restrict transcytosis. They also operate a repertoire of selective transporters and efflux pumps (GLUT1, P-glycoprotein, and others) that precisely control the movement of nutrients, metabolites, and xenobiotics between blood and brain (Paul et al., 2013).

Endothelial dysfunction, in the context of the immune-endothelial-neural axis, is best understood not as an all-or-none event but as a progressive phenotypic transition along a continuum (Wälchli et al., 2024). At one end lies the quiescent, protective phenotype described above; at the other lies overt structural disintegration of the vessel wall. Between these extremes, endothelial cells pass through at least two intermediate states that carry distinct functional consequences (Wälchli et al., 2024; Pober and Sessa, 2007). The first transition, from quiescence to mild activation, is reversible and occurs in response to subclinical systemic inflammation, modest hemodynamic stress, or early metabolic disturbance (Pober and Sessa, 2007). Endothelial cells begin to upregulate adhesion molecule expression and secrete low levels of chemokines, yet tight junction architecture remains largely intact, and BBB permeability is only minimally increased (Wälchli et al., 2024). This state, sometimes termed “endothelial priming,” does not by itself cause neurological symptoms. It does, however, lower the threshold at which a second hit can tip the system into overt pathology. Such hits include an acute infection, an ischemic episode, or a surge of systemic cytokines (Carvalho and Moreira, 2018). The primed state may persist for months or years before overt disease manifests. It is therefore, in principle, a detectable and potentially modifiable preclinical phase, analogous to hypertension or hyperglycemia in cardiovascular medicine (Vaitkevicius-Antão et al., 2025). Clinically, this stage may correspond to the asymptomatic phase of CSVD, in which white matter hyperintensities accumulate silently on MRI before cognitive decline becomes apparent (Wardlaw et al., 2019).

The second transition, from mild activation to overt endothelial injury, involves irreversible or slowly reversible structural changes (Yuan et al., 2020; Yang and Rosenberg, 2011). ROS accumulate, eNOS becomes uncoupled (reducing NO bioavailability while paradoxically increasing superoxide production), and junctional complexes undergo phosphorylation, internalization, or proteolytic degradation (Chen et al., 2010). The basement membrane thins or fragments, pericyte-endothelial coupling weakens, and the glycocalyx sheds (Patterson et al., 2022; Hamann et al., 1995). At this stage, BBB permeability rises substantially. Plasma proteins extravasate into the perivascular space, peripheral immune cells infiltrate the CNS parenchyma in appreciable numbers, and microthrombi form in the distal microcirculation (Martínez Santamaría et al., 2025). In ischemic stroke, this transition occurs acutely; in CSVD and VCID, it unfolds over years (Crumpler et al., 2021).

A defining feature of this continuum is that each step amplifies the next. Mildly primed endothelium recruits more circulating inflammatory cells. Once within the perivascular compartment, these cells release cytokines and matrix metalloproteinases (MMPs) that further degrade junctional proteins and basement membrane components. This accelerates the transition from activation to injury (Yang et al., 2019). In this sense, endothelial dysfunction is not merely a downstream consequence of neuroinflammation but a positive feedback driver. This distinction has therapeutic implications, because intervening at the priming stage could theoretically prevent the cascade from reaching the point of structural damage (Chen T. et al., 2024; Saharinen et al., 2017). The following section examines how this phenotypic continuum manifests across specific disease contexts.

### 2.2 Endothelial pathology across disease spectrums

Clinical and experimental evidence implicates cerebrovascular endothelial dysfunction in every major category of neurological disease. Its contribution, however, differs substantially between conditions in nature, magnitude, tempo, and dominant mechanism.

#### 2.2.1 Ischemic stroke

The ischemia-reperfusion sequence imposes maximal acute stress on the cerebrovascular endothelium (Sandoval and Witt, 2008). During ischemia, endothelial cells endure hypoxia, ATP depletion, and acidosis. eNOS activity declines, NADPH oxidases (NOX) are activated, and a burst of ROS shifts the endothelium from a vasodilatory to a vasoconstrictive, pro-oxidative state (Chen et al., 2010; Li et al., 2022). Upon reperfusion, the sudden return of pressure, shear stress, and oxygen-derived free radicals into a fragile microvascular bed accelerates tight-junction degradation, leading to rapid BBB destabilization (Krishnamoorthy et al., 2023). Adhesion molecule and tissue factor expression surge, driving leukocyte adhesion and microthrombus formation. Radiologically, these events manifest as vasogenic edema, contrast extravasation, or hemorrhagic transformation; in the circulation, sICAM-1, von Willebrand factor (vWF), and MMP-9 rise in proportion to BBB disruption (Krishnamoorthy et al., 2023). Animal studies support a causal role: blocking adhesion molecules or inhibiting MMP activity attenuates both BBB leakage and hemorrhagic complications. The endothelium, in this context, is both victim and amplifier: its structural status directly shapes the balance between tissue salvage and secondary injury (Candelario-Jalil et al., 2022; Anrather and Iadecola, 2016; Mathiesen et al., 2021).

#### 2.2.2 ICH and CSVD

In ICH, erythrocyte lysis within the hematoma releases hemoglobin and free iron, which drives hydroxyl radical production via the Fenton reaction and inflicts severe oxidative injury on perihematomal endothelial cells (Xi et al., 2006; Keep et al., 2012). Activated microglia and infiltrating macrophages, recruited to clear blood degradation products, secrete cytokines and MMPs that further disrupt tight junctions and the basement membrane. The result is persistent perihematomal BBB leakage and edema, a lesion whose resolution or expansion depends largely on the local endothelial response. CSVD, by contrast, is chronic and insidious (Hong et al., 2025). Hypertension and arterial stiffness produce arteriolar hyalinosis, luminal narrowing, and amplified pulsatile stress on the microvascular endothelium. Aging and metabolic disease add layers of chronic inflammation and oxidative damage (Iadecola and Davisson, 2008; Xu M. et al., 2024). MRI fingerprints, including white matter hyperintensities, lacunar infarcts, and cerebral microbleeds, reflect repeated cycles of endothelial fragility and BBB microdisruption (Wardlaw et al., 2019, 2013). Pathologically, fibrinoid necrosis of small venules and capillaries, perivascular space enlargement, and subendothelial iron deposition are common endpoints (Brown and Thore, 2011). Despite their different timescales, ICH and CSVD share a single pathological logic: hemodynamic or blood-derived burden, endothelial inflammation and structural injury, BBB breakdown, and perivascular remodeling (Pantoni, 2010).

#### 2.2.3 MS and NMOSD

In neuroimmune diseases, the endothelium is not merely a bystander damaged by inflammation but the gate through which the disease process enters the CNS (Engelhardt and Ransohoff, 2005). In MS, activated myelin-reactive T and B lymphocytes, primed in the periphery, cross the BBB via endothelial adhesion molecules and chemokine gradients (Engelhardt and Ransohoff, 2012). Once within the perivascular space, these cells initiate demyelination and axonal injury (Lassmann, 2018). Local cytokine release (IFN-γ, IL-17, GM-CSF) and oxidative stress reciprocally enhance endothelial activation. This lowers the functional threshold of the BBB and perpetuates a self-sustaining cycle of immune infiltration and vascular injury. NMOSD follows a related but distinct path: anti-AQP4 antibodies, binding to AQP4 on astrocytic endfeet, trigger complement-mediated injury that secondarily disrupts the endothelial-astrocyte barrier (Papadopoulos and Verkman, 2012; Jarius and Wildemann, 2010). In both conditions, the endothelial component of the NVU is a decisive determinant of disease activity and relapse severity.

#### 2.2.4 VCID and AD

Chronic endothelial dysfunction produces a more insidious form of injury: gradual microcirculatory failure that erodes neural networks over years (Schäfer et al., 2013). In VCID and mixed dementia, widespread white matter lesions, lacunar infarcts, and cerebral microbleeds on MRI are accompanied by carotid intima-media thickening, arterial stiffness, and reduced cerebrovascular reactivity (CVR). These are markers of long-standing endothelial compromise (Herzog et al., 2025). The mechanistic chain is clear: reduced NO bioavailability, increased endothelin-mediated vasoconstriction, and chronic endothelial inflammation blunt microvascular responsiveness to CO_2_, blood pressure, and functional demand. This chronically uncouples neural activity from blood supply (Kisler et al., 2017). In AD, the core pathological process is intraneuronal protein aggregation together with neuroinflammation intrinsic to the brain. Vascular and endothelial changes act as contributing and amplifying factors rather than as the primary driver. Cerebral amyloid angiopathy (CAA) deposits Aβ within small arteries and capillaries, injuring endothelial cells, reducing CBF, and provoking recurrent microhemorrhages (Greenberg et al., 2020). BBB dysfunction simultaneously impairs Aβ clearance and admits peripheral inflammatory mediators, further activating microglia and aggravating synaptic damage. In a subset of patients, hippocampal BBB leakage and changes in plasma and CSF endothelial markers are detectable at the mild cognitive impairment stage, before structural atrophy becomes apparent. Whether these changes are causal or consequential remains to be determined (Montagne et al., 2015; Nation et al., 2019).

#### 2.2.5 Parkinson's disease

Parkinson's disease (PD) is included in the framework proposed here, but its evidence base is appreciably weaker and less consistent than that of Alzheimer's disease. This difference should be kept in view when the two conditions are compared. Biofluid studies of Lewy body spectrum diseases suggest blood-brain barrier disruption, including a meta-analysis of the CSF-to-serum albumin quotient; the pooled effect is, however, highly heterogeneous between cohorts. Increased barrier permeability in PD is plausible but not uniformly demonstrable (Wong et al., 2022). Consistent with an endothelial contribution, clinical cohorts show impaired flow-mediated dilation and raised homocysteine in PD, with the degree of impairment relating to disease severity (Yoon et al., 2014). Peripheral immune activation is also measurable in patients: blood T-cell and monocyte phenotypes correlate both with central inflammatory signals measured by PET and with motor severity scores (Greenland et al., 2025). At tissue level, region-specific vascular changes, including loss of pericytes and altered perivascular spaces that track with Lewy body stage, have been described in the substantia nigra and medial temporal lobe of PD brains. This indicates that nigrostriatal vulnerability has a vascular component (Dik et al., 2026). The link between α-synuclein pathology and vascular inflammation nevertheless remains largely correlative: whether α-synuclein aggregation drives endothelial injury, or whether vascular dysfunction favors its propagation, is not established. PD is therefore presented here as a partially supported example of the axis rather than as an equally established one.

A common thread emerges across these conditions. Regardless of whether the initial insult is ischemic, hemorrhagic, immune-mediated, or degenerative, the cerebrovascular endothelium is both the sensor that detects the disturbance and the translator that converts it into a CNS-level pathological signal. The dominant phenotype (catastrophic BBB collapse, microthrombotic occlusion, or chronic hypoperfusion) depends on the disease context, but the underlying logic remains the same (Herzog et al., 2025; Xiao et al., 2022). In disorders where endothelial dysfunction is causally implicated rather than merely concomitant, this shared vulnerability makes the endothelium a compelling therapeutic target.

## 3 Mechanisms of immune-endothelial crosstalk in neuroinflammation

### 3.1 Innate immune cell-endothelial interactions

Innate immune cells are the first responders at the cerebrovascular interface. Their interactions with brain endothelial cells, through adhesion, transmigration, and effector molecule release, determine whether the initial response to injury resolves or escalates (Ley et al., 2007; Engelhardt and Ransohoff, 2005).

#### 3.1.1 Neutrophils

In stroke, infection, and systemic inflammation, circulating neutrophils are rapidly recruited to the cerebral microvasculature by IL-1β, TNF-α, and CXCL chemokines (Xie et al., 2023; Gelderblom et al., 2009). Their endothelial engagement follows the familiar cascade of selectin-mediated rolling, integrin (LFA-1, Mac-1)-dependent firm adhesion to ICAM-1 and VCAM-1, and protease-assisted transmigration through paracellular or transcellular routes (Phillipson and Kubes, 2011). The acute-phase function of neutrophil recruitment (pathogen clearance and debris removal) is essential, but the brain tolerates this response poorly. Excessive or persistent neutrophil infiltration overwhelms local antioxidant and antiprotease defenses, as activated neutrophils release ROS, elastase, myeloperoxidase, and inflammatory mediators that cumulatively degrade the glycocalyx, tight junctions, and basement membrane (Dang et al., 2013; Stowe et al., 2009). Human evidence for this mechanism is direct for intravascular neutrophil accumulation after stroke. The contribution of individual effector molecules, by contrast, rests largely on animal and *in vitro* work (Table 2).

**Table 2:** Evidence hierarchy for key immune-endothelial mechanisms.

Mechanism	Human interventional or genetic evidence	Human observational evidence (tissue, CSF, imaging, blood)	Animal models	*In vitro* systems
Neutrophil recruitment and NET formation	—	Human stroke thrombi (Laridan et al., 2017)	Yes	Yes
Monocyte and macrophage recruitment	—	Human stroke and CSVD tissue; circulating monocyte subsets	Yes	Yes
Endothelial antigen presentation	—	Reported in MS lesions but inconsistent (Traugott, 1987; Sasaki and Nakazato, 1992)	Yes	Yes
Th1 and Th17 recruitment	Anti-integrin therapy in MS (natalizumab) (Polman et al., 2006)	Human MS blood and CSF (van Langelaar et al., 2018)	Yes	Yes
Regulatory T-cell effects	—	Human stroke; circulating regulatory T-cell frequencies	Yes	Yes
CD8-mediated endothelial injury	—	Human CNS vasculitis and cerebral venous thrombosis histology	Yes	Yes
Complement activation and MAC deposition	—	Human NMOSD autopsy tissue (Takai et al., 2021)	Yes	Yes
Endothelial-microglial signaling	—	Human brain transcriptomics; perivascular macrophages (Brioschi et al., 2026)	Yes	Yes
Junctional remodeling and permeability	—	Human MS and CSVD tissue; CSF and imaging markers	Yes	Yes
Glycocalyx shedding	—	Human sepsis and critical illness	Yes	Yes
Endothelial death programs (apoptosis, pyroptosis, ferroptosis)	—	Limited human evidence; shared regulators documented *in vitro* (Zheng et al., 2022)	Yes	Yes

Human interventional or genetic evidence refers to patient studies that directly modify the pathway, or to human genetic evidence; human observational evidence comprises post-mortem tissue, cerebrospinal fluid, imaging, and circulating markers. “Yes” indicates that the mechanism has been demonstrated in the corresponding system. The table is intended to make differences in the strength of evidence visible rather than to imply uniform support. Several mechanisms rest predominantly on animal and *in vitro* work, and these are identified as priorities for human study in Section 6.

#### 3.1.2 Neutrophil extracellular traps (NETs)

NETs are web-like structures of extruded nuclear chromatin studded with granule proteins, released by highly activated or dying neutrophils (Brinkmann et al., 2004). Within cerebral microvessels, NETs serve as a scaffold for leukocytes, platelets, and fibrin, promoting immunothrombosis that further compromises an already vulnerable microcirculation (Fuchs et al., 2010). Their embedded proteases and cationic proteins directly attack the endothelial glycocalyx and plasma membrane (Xu et al., 2009; Saffarzadeh et al., 2012). In ischemic stroke reperfusion injury and cerebral venous thrombosis, intravascular NET accumulation correlates with hemorrhagic transformation, infarct expansion, and poor outcome. This links a single innate immune effector mechanism directly to the worst clinical complications of endothelial failure (Jin et al., 2022; Di et al., 2025). NET formation has been demonstrated in human stroke thrombi, so this mechanism is supported by human observational evidence as well as by animal models (Table 2).

#### 3.1.3 Monocytes, macrophages, and microglia

Monocytes enter the CNS later than neutrophils, primarily through the CCL2/CCR2 and CCL5/CCR5 chemokine axes (Chen T. et al., 2024; Conductier et al., 2010; Mildner et al., 2009). Endothelial cells and perivascular glia secrete CCL2, creating gradients that attract CCR2^+^ monocytes to adhere, transmigrate, and differentiate into perivascular macrophages (Deshmane et al., 2009; Faraco et al., 2016). In the subacute and chronic phases of ICH, CSVD, and VCID, perivascular macrophage density correlates with basement membrane disruption, plasma protein extravasation, and white matter injury. This supports their role as amplifiers of sustained endothelial injury (Lapenna et al., 2018). These cells are not unidirectional agents of damage: they also phagocytose debris and blood degradation products, and under resolving conditions can adopt phenotypes that promote vascular repair (Lan et al., 2017). The balance between injury and repair, however, tilts toward damage when inflammation persists.

Microglia, the resident innate immune cells of the CNS, engage in a particularly complex bidirectional relationship with the endothelium. Their ramified processes continuously surveil the perivascular microenvironment, detecting DAMPs (ATP, HMGB1) and PAMPs that reach the perivascular space through a compromised BBB (Nimmerjahn et al., 2005; Davalos et al., 2005). Upon activation, microglia migrate toward damaged vessels and release cytokines and growth factors that may transiently stabilize the vascular wall. Prolonged stimulation, however, locks them into a pro-inflammatory state, chronically releasing ROS, NO, TNF-α, and IL-1β that progressively erode endothelial barrier function and vasoregulatory capacity (Kobeissy and Salzet, 2026; da Fonseca et al., 2014). This interaction is not one-directional: endothelial cells secrete CX3CL1 and TGF-β, which under physiological conditions restrain microglial activation and promote a surveillant, homeostatic phenotype (Cardona et al., 2006; Sheridan and Murphy, 2013). Dysregulation of this endothelium-microglia axis, therefore, produces not only local BBB failure but also broader propagation of neuroinflammation. This manifests clinically as secondary lesion expansion after stroke, worsening white matter injury, and progressive cognitive decline (Luo and Qiao, 2026; Wang et al., 2026).

#### 3.1.4 Innate immune signaling pathways

Downstream of cellular contacts, pattern-recognition receptors and the complement system serve as molecular amplifiers (He et al., 2026; Hanke and Kielian, 2011; Orsini et al., 2014). TLR2 and TLR4 on endothelial cells and perivascular immune cells recognize DAMPs and PAMPs, activating NF-κB and IRF pathways that drive adhesion molecule upregulation and cytokine secretion (Hanke and Kielian, 2011; Nagyoszi et al., 2010). The NLRP3 inflammasome, assembled in endothelial cells, macrophages, and microglia, processes IL-1β and IL-18 into their active forms and can trigger pyroptotic cell death. This releases additional DAMPs that perpetuate the inflammatory loop (Walsh et al., 2014; Guo et al., 2015). The complement cascade generates C3a and C5a, potent chemoattractants for neutrophils and monocytes, while membrane attack complex (MAC) deposition causes sublethal endothelial injury or outright lysis (Orsini et al., 2014; Ricklin et al., 2010). These pathways are essential for host defense but, in the CNS, operate with limited tolerance. Once uncontrolled, they rapidly convert the endothelial interface from a protective barrier into a conduit for self-sustaining neuroinflammation (Galea, 2021; Ricklin et al., 2010).

### 3.2 Adaptive immune cell-endothelial interactions

Where innate immunity provides the initial, rapid response at the cerebrovascular interface, adaptive immunity operates with greater specificity and immunological memory. It shapes the subacute and chronic trajectory of neuroinflammation and microvascular pathology (Anrather and Iadecola, 2016; Iadecola and Anrather, 2011).

#### 3.2.1 CD4^+^ T cells

T-cell entry into the CNS depends on prior peripheral antigen activation and upregulation of specific integrins and chemokine receptors (Engelhardt and Ransohoff, 2012; Wilson et al., 2010). Under inflammatory conditions, endothelial cells upregulate ICAM-1, VCAM-1, and selectins while secreting chemokines such as CXCL9/10/11 and CCL20. This creates a vascular surface that preferentially recruits particular T-cell subsets to defined brain regions. It thereby contributes to the spatial heterogeneity of immune infiltration across diseases (Sallusto et al., 2012; Wilson et al., 2010; Reboldi et al., 2009). The endothelium is not merely a passive filter in this process; its adhesion molecule and chemokine repertoire actively regulate the local T-cell composition (Ley et al., 2007; Ransohoff et al., 2003).

Among CD4^+^ subsets, Th1 and Th17 cells are the principal endothelial agonists. Th1 cells, through IFN-γ and TNF-α, induce endothelial MHC class II and costimulatory molecule expression. This effectively transforms the vascular wall from a low-visibility barrier into an atypical antigen-presenting surface (Pober et al., 2017; Wheway et al., 2013). IFN-γ simultaneously amplifies ICAM-1/VCAM-1 expression, recruiting yet more Th1 cells, while impairing NO signaling and barrier stability (Sonar and Lal, 2018; Pober and Tellides, 2012). Th17 cells act through IL-17A/F, IL-22, and GM-CSF. IL-17 directly triggers tight junction redistribution and cytoskeletal remodeling in brain endothelial cells, increases paracellular permeability, and induces secretion of CXCL1/2 and G-CSF that recruit neutrophils. This establishes a Th17-endothelial-neutrophil axis that drives inflammation deeper into white and gray matter, a mechanism well characterized in EAE and increasingly implicated in CSVD and vasculitis lesions (Kebir et al., 2007, 2009).

#### 3.2.2 Regulatory T cells (Tregs)

Through IL-10, TGF-β, and CTLA-4-dependent mechanisms, Tregs suppress endothelial NF-κB signaling, downregulate adhesion molecule expression, and promote eNOS activity and tight-junction recovery. These mechanisms exert both vascular-protective and neuroprotective effects in post-stroke and chronic ischemia models (Liesz et al., 2009; Li et al., 2013). The Th1/Th17-Treg imbalance thus functions as an immunological barometer of the neuroinflammatory milieu, with the endothelium serving simultaneously as its target and its regulator (Hu et al., 2014; Kleinewietfeld and Hafler, 2013).

#### 3.2.3 CD8^+^ T cells

Beyond helper functions, CD8^+^ cytotoxic T cells can directly kill endothelial cells (Suidan et al., 2008; Nitcheu et al., 2003). Upon recognizing peptide-MHC class I complexes on the endothelial surface, activated CD8^+^ T cells induce apoptosis through perforin, granzymes, or Fas/FasL-TRAIL death signaling. This produces focal capillary wall rupture and microhemorrhage (Pober and Tellides, 2012; Biedermann and Pober, 1998). Ultrastructural evidence from immune-mediated cerebral vasculitis and cerebral venous sinus thrombosis shows CD8^+^ T cells within the vascular wall in direct contact with endothelial cells. This confirms that adaptive cytotoxicity can be a primary driver of vascular injury rather than a secondary bystander effect (Salvarani et al., 2012; Hajj-Ali et al., 2011; Salvarani et al., 2007). This mechanism is supported by human histological evidence from CNS vasculitis and cerebral venous thrombosis, while the corresponding functional experiments remain largely preclinical (Table 2).

#### 3.2.4 B cells and antibodies

B cells contribute to cerebrovascular pathology through multiple routes. As antigen-presenting cells, they sustain T-cell activation within meningeal and perivascular tertiary lymphoid-like structures. Their secreted cytokines, including IL-6 and GM-CSF, influence endothelial activation at a distance (Galea, 2021; Tanaka et al., 2014; Becher et al., 2016). The most direct endothelial injury, however, comes from autoantibodies. Anti-endothelial cell antibodies (AECAs), detectable in primary CNS vasculitis and related conditions, bind endothelial surface antigens, activate complement, and induce dysfunction or death through Fc receptor-mediated cytotoxicity (Praprotnik et al., 2001). Immune complex deposition within small vessel walls triggers the classical complement cascade, recruiting neutrophils and monocytes and producing leukocytoclastic vasculitis-like changes, fibrinoid necrosis, and luminal occlusion (Jennette and Falk, 1997; Sunderkötter et al., 2018). Even in AD, emerging evidence suggests that autoantibodies against neuronal or vascular components may accelerate disease progression in a subset of patients. The clinical significance of this finding, however, remains to be fully defined. Across Sections 3.1 and 3.2, the strength of evidence differs substantially between mechanisms. Junctional remodeling, perivascular immune accumulation, and complement deposition are documented in human tissue. By contrast, endothelial antigen presentation and several aspects of endothelial-microglial signaling rest predominantly on animal and *in vitro* work (Table 2).

Across these diverse pathways, a consistent logic emerges: adaptive immune cells are not merely peripheral participants in neurovascular disease but active shapers of endothelial phenotype. The endothelium reciprocally determines which adaptive effectors gain access to the CNS. Understanding this bidirectional coupling (which immune cell subsets gain access, under what conditions, and with what consequences) is therefore a prerequisite to any disease-specific therapeutic strategy (Figure 2).

**Figure 2:**
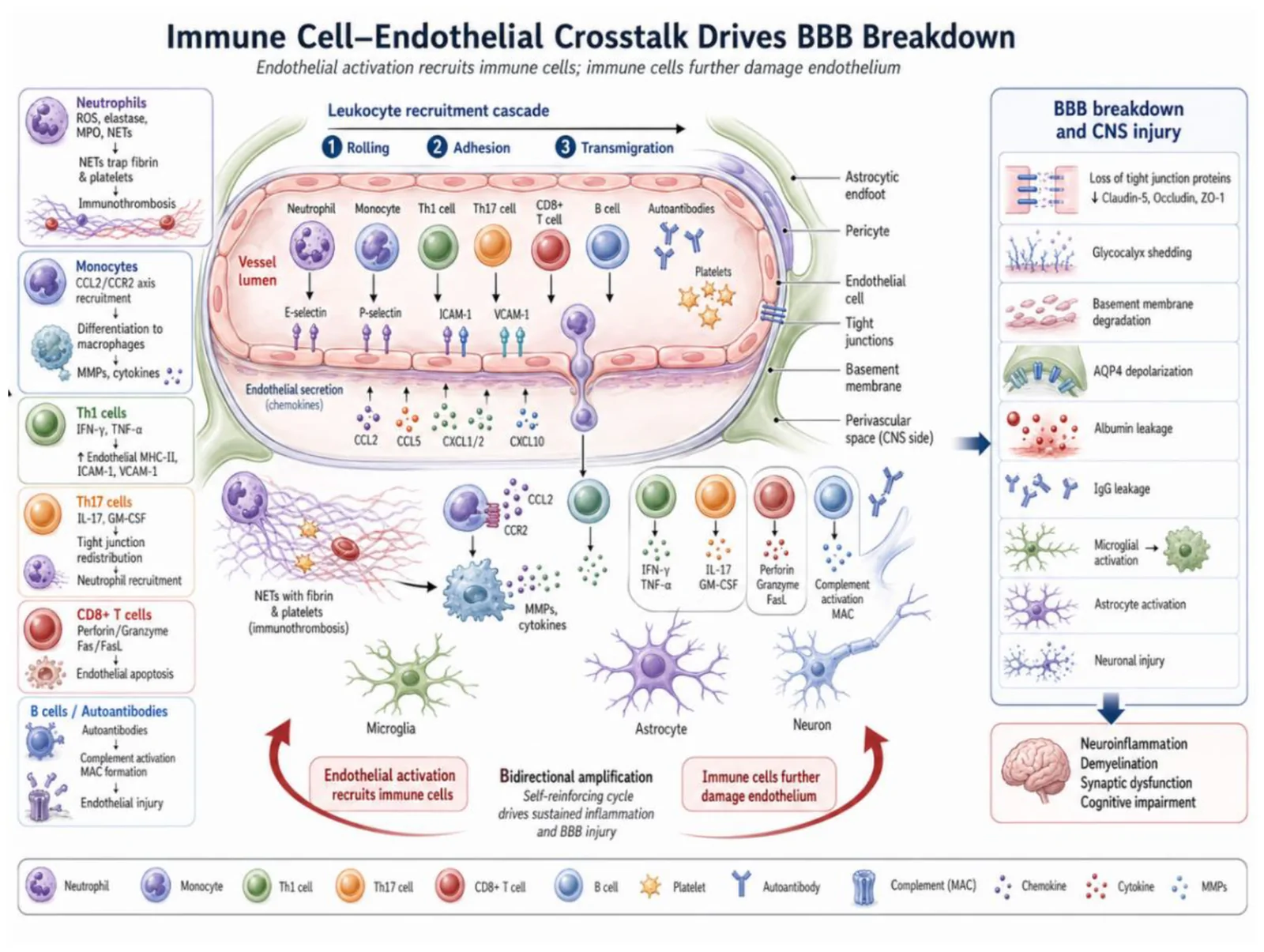
Immune cell-endothelial crosstalk drives blood-brain barrier breakdown. Left panel: Six immune cell subsets and their pathogenic effector molecules—neutrophils (which drive immunothrombosis through ROS, elastase, MPO, and NETs), monocytes (recruited via CCL2/CCR2 and differentiating into perivascular macrophages that secrete MMPs and cytokines), Th1 cells (which upregulate MHC-II, ICAM-1, and VCAM-1 through IFN-γ and TNF-α), Th17 cells (which induce tight junction redistribution and neutrophil recruitment through IL-17 and GM-CSF), CD8^+^ T cells (which trigger endothelial apoptosis through perforin, granzyme, and Fas/FasL), and B cells/autoantibodies (which cause endothelial injury through complement activation and membrane attack complex formation). Central panel: Cross-sectional view of a cerebral microvessel depicting the three-step leukocyte recruitment cascade: (1) selectin-mediated rolling, (2) integrin-dependent firm adhesion to ICAM-1 and VCAM-1, and (3) transendothelial migration (diapedesis). Endothelial cells secrete chemokines (CCL2, CCL5, CXCL1/2, CXCL10) that guide immune cell trafficking. Perivascular events include NET-mediated immunothrombosis, macrophage-derived MMP secretion, T-cell-mediated glial activation, and complement-dependent neuronal injury. Right panel: Nine progressive pathological consequences of sustained immune-endothelial interaction—loss of tight junction proteins (claudin-5, occludin, ZO-1), glycocalyx shedding, basement membrane degradation, AQP4 depolarization, albumin and IgG leakage, microglial activation, astrocyte activation, and neuronal injury—culminating in neuroinflammation, demyelination, synaptic dysfunction, and cognitive impairment. Bottom: Two red curved arrows forming a self-reinforcing cycle emphasize that endothelial activation recruits immune cells, which in turn further damage the endothelium, driving sustained inflammation and BBB injury.

### 3.3 Molecular networks: amplification loops

The selectin-integrin-adhesion molecule axis and its associated cytokine/chemokine network have been described in preceding sections; here we focus on their emerging characteristics as a self-amplifying signaling system. The core positive feedback loop operates as follows. Peripheral or focal inflammation elevates TNF-α, IL-1β, IL-6, and IFN-γ. Endothelial cells sense these signals through NF-κB, JAK/STAT, and p38 MAPK pathways and upregulate E-/P-selectins, ICAM-1, VCAM-1, and chemokines (CCL2, CCL5, CXCL1/2, CXCL10) (Ley et al., 2007; Pober and Sessa, 2007; Pober, 2002). Recruited immune cells, including neutrophils, monocytes, and T cells, release additional cytokines, proteases, and ROS that further intensify endothelial activation. This creates a self-perpetuating loop in which inflammation activates the endothelium, endothelial activation recruits more immune cells, and immune recruitment sustains additional inflammation (Varatharaj and Galea, 2017; Nathan, 2002).

Within this loop, specific chemokine axes confer spatial and temporal selectivity. CCL2, produced jointly by endothelial cells, astrocytes, and microglia, establishes a vascular-to-parenchymal gradient that recruits CCR2^+^ monocytes and memory T cells (Deshmane et al., 2009; Mildner et al., 2007; Shi and Pamer, 2011). CXCL10, induced by IFN-γ, preferentially attracts Th1 cells and NK cells via CXCR3, a pathway linked to lesion activity in MS and viral encephalitis. CXCL1/2-CXCR2 guides neutrophils, while CCL5-CCR5 sustains secondary T-cell and monocyte recruitment. These axes are layered in time and superimposed in space, meaning that different immune lineages accumulate at different phases and in different microenvironments (Gelderblom et al., 2009; Shi and Pamer, 2011; Mrdjen et al., 2018).

Critically, the loop does not remain confined to the vascular wall. Activated microglia and astrocytes, responding to vascular-derived cytokines and chemokines, secrete their inflammatory mediators that feed back to the endothelium. Injured neurons release DAMPs that stimulate endothelial TLR and NLR signaling. The result is a self-sustaining inflammatory circuit that extends across the entire NVU.

Whether this circuit resolves or escalates depends on the balance between positive amplification and negative feedback. IL-10 and TGF-β, produced by Tregs, M2-like macrophages, and, under some conditions, endothelial cells themselves, suppress NF-κB signaling, downregulate adhesion molecule expression, and promote junctional recovery (Sweeney et al., 2019; Lin et al., 2018; Walshe et al., 2009). Specialized pro-resolving mediators (SPMs), including resolvins and protectins derived from omega-3 fatty acids, actively terminate neutrophil recruitment and promote macrophage efferocytosis. When these brakes fail (because the inflammatory stimulus is too strong, too persistent, or too temporally dispersed), the amplification loop overrides negative feedback. The endothelium then progresses from a reversible activated state to structural disruption (Sweeney et al., 2018; Zlokovic, 2008; Iadecola, 2013). This tipping point between resolution and chronicity provides a rational basis for therapeutic strategies that target not a single cytokine but the architecture of the loop itself. Such strategies may block adhesion molecules (e.g., natalizumab against VLA-4 in MS), antagonize specific cytokines (e.g., anti-IL-6R or anti-TNF agents), or boost endogenous resolving pathways (Polman et al., 2006; Serhan, 2014; Traboulsee et al., 2020). The molecular loops described in this section are supported mainly by mechanistic studies in animals and cell systems. Direct human interventional evidence is at present limited to therapies acting on single nodes of the loop, such as adhesion molecule blockade and cytokine antagonism. Their partial clinical efficacy is consistent with, but does not prove, the circuit model proposed here (Table 2).

### 3.4 Blood-brain barrier structural remodeling and permeability

BBB failure is not a single event but the cumulative result of coordinated structural changes at four levels. These are the intercellular junctions, the luminal glycocalyx, the basement membrane, and astrocytic endfeet.

#### 3.4.1 Tight and adherens junctions

Under inflammatory signaling (TNF-α, IL-1β, VEGF, Ang-2, ROS), Rho/ROCK, PKC, and Src pathways drive phosphorylation, ubiquitination, and internalization of claudin-5, occludin, and ZO-1, uncoupling them from the actin cytoskeleton (Hawkins and Davis, 2005; Stamatovic et al., 2008). VE-cadherin and β-catenin at adherens junctions undergo similar redistribution, weakening inter-endothelial adhesion and permitting cytoskeletal tension to widen paracellular gaps (Giannotta et al., 2013; Dejana et al., 2008). The morphological consequence (visible on immunofluorescence as a shift from continuous belt-like to punctate junctional staining) directly lowers paracellular resistance. This allows albumin, immunoglobulins, and other plasma macromolecules to leak into the perivascular space (Knowland et al., 2014; Shen et al., 2011).

#### 3.4.2 The endothelial glycocalyx

This gel-like layer of glycosaminoglycans, proteoglycans, and glycoproteins coats the luminal endothelial surface and forms an electrostatic and mechanical barrier that buffers shear stress, sequesters anticoagulant factors, and limits leukocyte and platelet adhesion (Reitsma et al., 2007; Nieuwdorp et al., 2005; Salmon and Satchell, 2012). During neuroinflammation, MMPs, heparanase, and hyaluronidase fragment the glycocalyx into sparse, discontinuous remnants. The consequences are twofold: underlying adhesion molecules and receptors become exposed, making the endothelial surface more permissive for leukocyte adhesion. The loss of shear-buffering capacity creates local turbulence that favors microthrombus formation (Mulivor and Lipowsky, 2002; Constantinescu et al., 2003; Uchimido et al., 2019).

#### 3.4.3 The basement membrane

Composed of type IV collagen, laminin, and heparan sulfate proteoglycans, the basement membrane anchors pericytes and astrocytic endfeet while providing a final physical barrier to immune cell migration (Thomsen et al., 2017; Baeten and Akassoglou, 2011). MMP-2 and MMP-9, released by endothelial cells, pericytes, macrophages, and microglia, degrade or focally rupture this structure (Candelario-Jalil et al., 2009; Rosenberg, 2002). Beyond removing the last physical barrier to parenchymal entry, basement membrane disruption releases matrix-sequestered growth factors and cytokines, induces perivascular gliosis and fibrosis, and alters interstitial fluid dynamics. These changes are particularly prominent in CSVD, VCID, and AD (Iadecola, 2017; Wardlaw et al., 2019; Iadecola et al., 2019).

#### 3.4.4 AQP4 and astrocytic endfeet

AQP4, expressed at high density on astrocytic endfeet, is the principal water channel governing brain water exchange and glymphatic clearance (Liao et al., 2024; Krings et al., 2025; Iliff et al., 2012). Under physiological conditions, AQP4 is tightly polarized to perivascular domains, where arterial pulsation drives clearance of interstitial waste (Nielsen et al., 1997; Mader and Brimberg, 2019; Kress et al., 2014). When BBB leakage exceeds compensatory capacity, AQP4-mediated water influx into astrocytes produces cell swelling and endfoot expansion that compresses the perivascular space. This further impairs microcirculation and glymphatic flow (Papadopoulos and Verkman, 2013; Haj-Yasein et al., 2011). Loss of AQP4 polarity (redistribution from endfeet to soma under inflammatory conditions) disrupts organized water exchange and makes edema more difficult to resolve (Mader and Brimberg, 2019; Fukuda and Badaut, 2012). In ischemic stroke, ICH, TBI, and NMOSD, marked vasogenic edema correlates with AQP4 depolarization and swollen endfeet. This positions AQP4 as both an emergency regulator of BBB failure and an active contributor to edema progression (Papadopoulos and Verkman, 2013; Manley et al., 2000).

Together, these four levels of remodeling transform the BBB from a high-resistance, selective barrier into a hyperpermeable interface. The permeability phenotype (whether manifest as subtle chronic leakage in CSVD or explosive disruption in stroke) is determined by which layers fail, in what order, and to what degree.

### 3.5 Oxidative stress, metabolic reprogramming, and cell death

Cerebrovascular endothelial cells are not merely passive targets of inflammatory injury. Through their own redox state, metabolic programs, and cell fate decisions, they actively shape the trajectory of neurovascular disease.

#### 3.5.1 ROS/RNS imbalance

Under physiological conditions, eNOS-derived NO maintains vasodilation while low-level mitochondrial ROS serve as signaling molecules (Chen et al., 2010; Schieber and Chandel, 2014; Kluge et al., 2013). Chronic exposure to vascular risk factors (hypertension, hyperglycemia, dyslipidemia) and inflammatory mediators (TNF-α, IL-1β, angiotensin II) is associated with a shift in this balance. NOX (NOX2, NOX4), xanthine oxidase, and uncoupled eNOS collectively surge superoxide production. Superoxide rapidly scavenges NO to form peroxynitrite, simultaneously depleting a vasoprotective mediator and generating a potent oxidant (Förstermann et al., 2017; Bedard and Krause, 2007). Mitochondrial membrane destabilization during ischemia-reperfusion adds a further ROS burst. The integrated consequences cascade across multiple domains. NO bioavailability falls, and vasoconstriction predominates; tight junction proteins undergo oxidative modification and mislocalization; adhesion molecule and tissue factor expression rises through ROS-driven NF-κB signaling; and BBB permeability increases (Förstermann et al., 2017; Pun et al., 2009). ROS/RNS imbalance thus functions as a hub that links endothelial dysfunction, microcirculatory failure, and neuroinflammation into a single pathogenic axis.

#### 3.5.2 Metabolic reprogramming

Endothelial cells normally rely predominantly on glycolysis rather than oxidative phosphorylation, a metabolic strategy that minimizes mitochondrial ROS and reserves oxygen for neurons (Eelen et al., 2018; De Bock et al., 2013). Under chronic inflammation, ischemia, or metabolic stress, HIF-1α, NF-κB, and mTOR signaling drive further glycolytic upregulation, increased lactate production, and suppression of mitochondrial oxidative phosphorylation (Eelen et al., 2018; De Bock et al., 2013). In the short term, this adaptation supports ATP generation under hypoxic conditions. Over time, however, mitochondrial dysfunction and impaired mitophagy transform mitochondria from regulatory organelles into additional ROS sources (Schieber and Chandel, 2014; Qu et al., 2022). Metabolic reprogramming also alters inflammatory phenotype. Lactate, acting via GPR81, sustains adhesion molecule and cytokine expression. Suppressed fatty acid oxidation and lipid droplet accumulation create substrates for lipid peroxidation; and the pentose phosphate pathway generates NADPH that feeds back into NOX activity (Eelen et al., 2018). The net effect is a feedforward cycle in which metabolic disturbance both drives and is driven by oxidative stress and inflammation (Pun et al., 2009; Reuter et al., 2010).

#### 3.5.3 Programmed cell death: apoptosis, pyroptosis, and ferroptosis

The fate of endothelial cells under sustained oxidative and metabolic stress is determined by which death pathway predominates, a choice biased by the local biochemical environment (Zheng et al., 2022). Classical apoptosis, triggered via Fas/FasL, TNF-α/TNFR, or mitochondrial injury, proceeds through caspase-3 activation to produce orderly cell dismantling (Elmore, 2007). Detachment of apoptotic endothelial cells leaves denuded basement membrane patches that promote platelet adhesion and microthrombus formation, accelerating BBB breakdown (Jurcau and Simion, 2021; Luo and Qiao, 2026).

Pyroptosis, driven by NLRP3 inflammasome-caspase-1 activation, cleaves gasdermin D (GSDMD) to form membrane pores, causing cells to swell, lyse, and release IL-1β, IL-18, and DAMPs (Shi et al., 2015; Liu et al., 2016). Because pyroptotic endothelial cells die while broadcasting an inflammatory payload, this pathway both causes abrupt structural BBB disruption and amplifies responses in surrounding microglia, pericytes, and immune cells. This links it to acute BBB collapse in stroke and focal inflammatory lesions in CSVD (Xu Y. et al., 2024; Song et al., 2026).

Ferroptosis, an iron-dependent lipid peroxidation pathway, is especially relevant in ICH and iron-overload conditions. Hemoglobin-derived free iron drives hydroxyl radical formation via the Fenton reaction, peroxidizing polyunsaturated fatty acids in endothelial membranes (Keep et al., 2012; Dixon et al., 2012). When the glutathione/GPX4 antioxidant system is overwhelmed, irreversible membrane damage ensues. This iron-lipid combustion directly injures endothelial cells and pericytes while sustaining a pro-inflammatory microenvironment (Zheng et al., 2022).

These three death modes are not mutually exclusive. They are better described as overlapping, context-dependent programs than as a continuum determined by stress intensity. They share upstream regulators, including reactive oxygen species, mitochondrial dysfunction, inflammasome activation, and iron handling. They can also occur simultaneously within heterogeneous vascular cell populations, including within the same vessel segment. Direct crosstalk has been documented in both directions. Caspase-3 and caspase-7, the executioners of apoptosis, cleave gasdermin D and thereby limit pyroptosis, whereas in the absence of gasdermin D, caspase-1 can activate caspase-3 and caspase-7 to drive apoptosis (Taabazuing et al., 2017). Conversely, caspase-3 cleavage of gasdermin E converts an apoptotic programme into a pyroptotic one (Wang et al., 2017). Lipid peroxidation products generated during ferroptosis can promote NLRP3 inflammasome activation in endothelial cells, and inflammasome activity in turn aggravates ferroptosis after cerebral ischemia-reperfusion (Xie et al., 2022; Wang et al., 2023). This relationship is not uniformly unidirectional, since 4-hydroxynonenal can suppress NLRP3-dependent pyroptosis under some conditions (Hsu et al., 2022). Because these programs share regulators and can be engaged simultaneously (Zheng et al., 2022), the stress-dependence scheme outlined above is best regarded as a heuristic simplification rather than as an established rule. It requires experimental verification in each disease context. That scheme assumes that mild oxidative stress favors apoptosis, intense inflammatory signaling favors pyroptosis, and iron overload favors ferroptosis. Which programme predominates shapes the clinical phenotype, whether diffuse microleakage, scattered microthrombi, or explosive BBB collapse with haemorrhagic transformation. Identifying the dominant mode in a given disease context may allow more selective therapeutic targeting.

## 4 A systems model of immune-endothelial-neural interactions in neurovascular disorders

### 4.1 Shared and disease-specific features

If the immune-endothelial-neural axis provides a meaningful conceptual framework, clinically distinct neurovascular disorders should exhibit overlapping pathological hallmarks while maintaining disease-specific features shaped by vascular caliber, inflammatory tempo, and regional vulnerability. Accumulating evidence supports this view.

#### 4.1.1 Shared hallmarks

Across ischemic stroke, ICH, CSVD, VCID, MS/NMOSD, and AD/PD, four features recur: (1) endothelial activation and phenotypic transition from protective to injurious; (2) BBB disruption, ranging from subtle chronic leakage to explosive acute breakdown; (3) microcirculatory failure, whether through microthrombosis, vasomotor dysregulation, or chronic hypoperfusion; and (4) sustained neuroinflammation involving innate and adaptive immune cells. In every case, these four elements form a self-reinforcing circuit rather than independent pathological events. This argument is supported by the observation that interventions targeting any single element (e.g., anti-adhesion therapy in MS, antihypertensive treatment in CSVD) produce only partial clinical benefit unless the underlying circuit is also addressed (Polman et al., 2006; Benavente et al., 2013).

#### 4.1.2 Disease-specific signatures

What differentiates one condition from another is not the presence or absence of endothelial-immune dysfunction but its tempo, vascular scale, and regional distribution (Table 3). Herein, we compare six pathological dimensions across four disease categories: ischemic stroke/intracerebral hemorrhage (Stroke/ICH), cerebral small vessel disease/vascular cognitive impairment (CSVD/VCID), multiple sclerosis/neuromyelitis optica spectrum disorder (MS/NMOSD), and Alzheimer's disease/Parkinson's disease (AD/PD). The rows of Table 3 differ in the strength of their support. The entries for predominant vessel involvement, predominant insult, key endothelial change, and regional vulnerability rest on direct human evidence. Those for inflammatory tempo and feedback direction, by contrast, are inferred from model systems and are proposed as part of the working model, as indicated in the “Basis” column. Despite differences in vascular caliber, inflammatory tempo, dominant insult, key endothelial changes, regional vulnerability, and feedback direction, all conditions share the fundamental architecture of the immune-endothelial-neural axis. This is a self-reinforcing circuit in which immune dysregulation activates the endothelium, BBB disruption admits further immune mediators, and neuroinflammation feeds back to worsen endothelial dysfunction.

**Table 3:** Proposed working model of shared and disease-specific features of the immune-endothelial-neural axis.

Dimension	Stroke/ICH	CSVD/VCID	MS/NMOSD	AD/PD	Basis
Predominant vessel involvement	Predominantly large and medium arteries; small penetrating vessels also involved	Penetrating arterioles, capillaries, venules	Small veins, perivenular spaces	Small arteries, capillaries	Evidence-based
Inflammatory tempo	Acute, days-weeks escalation, partial resolution	Chronic low-grade, years	Relapsing-remitting (MS) or acute (NMOSD)	Chronic low-grade, decades	Inferred
Predominant insult	Ischemia-reperfusion/blood degradation	Hemodynamic stress + metabolic burden	Antigen-specific immune attack	Protein aggregation + vascular aging	Evidence-based
Key endothelial change	Catastrophic BBB collapse, MMP surge	Gradual junctional erosion, microleakage	Immune-mediated activation, complement deposition	CAA-related stiffening, chronic leakage	Evidence-based
Regional vulnerability	Vascular territory-specific	Periventricular white matter, deep gray	Perivenular white matter (MS); optic nerve, spinal cord (NMOSD)	Hippocampus, medial temporal lobe (AD); substantia nigra (PD)	Evidence-based
Feedback direction	Strong systemic spillback (SIRS)	Weak feedback, insidious progression	CNS-to-peripheral immune amplification (EAE)	Bidirectional, low-amplitude	Proposed

This table shows a proposed working model rather than an established taxonomy, and its categories are hypotheses to be tested. Small penetrating vessels contribute to all four disease groups; the vessel classes listed indicate predominance rather than exclusion. Alzheimer's disease and Parkinson's disease are grouped because both are chronic proteinopathic disorders with a vascular component, but they differ in causative protein, immune mechanism, regional pattern of vulnerability, and the evidence base for Parkinson's disease is weaker, particularly for blood-brain barrier permeability. The “Basis” column indicates whether an entry is directly supported by human evidence (evidence-based), inferred from indirect or model-system evidence (inferred), or proposed as part of the working model (proposed).

#### 4.1.3 Implications for therapy

This shared-vs.-specific structure has direct translational consequences. On the shared side, strategies that stabilize endothelial phenotype, protect BBB integrity, or modulate immune-endothelial signaling have broad cross-disease potential. This explains, for example, why statins, RAAS blockers, and anti-inflammatory agents show benefit across multiple conditions despite their different etiologies (Wardlaw et al., 2019; Chen et al., 2023). On the specific side, successful intervention in individual diseases requires matching the therapeutic window to the inflammatory tempo, for example, acute immunotherapy in MS vs. chronic endothelial protection in CSVD. It also requires accounting for regional vulnerability, such as hippocampal BBB protection in AD vs. spinal cord-targeted approaches in NMOSD. The challenge for precision neurovascular medicine is to identify, for each patient, where along the axis the dominant pathology lies and which combination of shared and disease-specific interventions is most likely to restore equilibrium (Petersen et al., 2023).

### 4.2 Experimental models and translational tools

Testing the immune-endothelial-neural axis requires integrated platforms that resolve interactions across spatial, temporal, and cellular scales.

#### 4.2.1 *In vitro* systems

BBB monolayer and Transwell co-culture models, incorporating brain endothelial cells, pericytes, and astrocytes, remain the standard for initial screening of adhesion molecule blockade, signaling pathway inhibitors, and endothelial-protective agents. Barrier integrity is assessed by transendothelial electrical resistance and tracer permeability (Shamul et al., 2025; Jagtiani et al., 2022). While architecturally simple, these systems lack physiological shear stress and three-dimensional vascular geometry, limiting their ability to model leukocyte rolling and transmigration.

Microfluidic BBB-on-a-chip platforms address these limitations by incorporating endothelial cells, pericytes, and astrocytes into micrometer-scale channels with controlled flow, shear stress, and chemical gradients. They enable direct visualization of leukocyte adhesion, transendothelial migration, and microthrombus formation. Human iPSC-derived brain endothelial cells and vascularized brain organoids extend this approach further. They provide human-specific contexts for studying genetic susceptibility and chronic inflammation, though current models still face limitations in vascular maturation and immune component completeness (Lippmann et al., 2012).

#### 4.2.2 Animal models

*In vivo* models remain indispensable when hemodynamics, BBB integrity, immune trafficking, and neurological function must be assessed simultaneously. Middle cerebral artery occlusion (MCAO) is the standard for ischemia-reperfusion injury, reproducing endothelial activation, BBB breakdown, leukocyte infiltration, and edema across acute and chronic phases. Collagenase injection and autologous blood infusion model ICH, enabling study of blood degradation products and iron overload on the microvascular endothelium. CSVD and VCID are modeled through spontaneously hypertensive rats, bilateral common carotid artery stenosis, and high-fat diet models that recapitulate gradual endothelial activation, microthrombosis, and white matter injury. Experimental autoimmune encephalomyelitis (EAE) remains the reference model for neuroimmune disorders, reproducing antigen-specific T/B-cell infiltration, demyelination, and BBB disruption (Constantinescu et al., 2011; Kaiser et al., 2014).

Cell type-specific genetic tools, including Tie2-Cre or Cdh5-CreER for endothelial cells, LysM-Cre for monocyte-macrophages, CX3CR1-Cre for microglia, and CD4-Cre for T cells, allow causal dissection of individual molecular contributions (Kilani et al., 2019). These models are particularly valuable for studying multi-organ crosstalk in the context of an intact organism, but their translational relevance is constrained by species-specific differences from human pathology.

#### 4.2.3 Analytical technologies

Two-photon intravital microscopy through chronic cranial windows enables real-time observation of leukocyte rolling, adhesion, transmigration, BBB leakage, and microthrombus formation in living animals (Byun et al., 2022). When combined with neuronal calcium imaging or blood flow velocity measurement, immune-endothelial-neural activity can be mapped within a single microscopic field.

At the molecular level, single-cell RNA sequencing (scRNA-seq) and multimodal single-cell approaches define the state of endothelial cells, pericytes, astrocytes, microglia, and infiltrating immune cells across disease stages. These approaches distinguish protective from injurious endothelial subpopulations and identify gene modules associated with BBB stability and immune recruitment. Spatial transcriptomics and spatial proteomics preserve tissue architecture while resolving regional heterogeneity in perivascular inflammatory zones and lesion edges.

#### 4.2.4 Toward systems-level models

What has been achieved. Multi-omics integration, combining transcriptomic, proteomic, metabolomic, lipidomic, and imaging-derived data mapped onto defined cell types and anatomical regions, has begun to enable the construction of quantitative interaction networks for the immune-endothelial-neural axis (Sibilio et al., 2025). Single-cell atlases of the human brain vasculature define endothelial and mural cell states across development, adulthood, and disease (Wälchli et al., 2024); single-cell maps of brain macrophages and border-associated populations resolve the myeloid compartment (Orsini et al., 2014); and spatial transcriptomic approaches localize perivascular inflammatory zones and lesion edges within intact tissue architecture. These resources are descriptive and hypothesis-generating: they provide the input data for a systems model rather than the model itself.

What remains aspirational. A parameterized, prospectively validated predictive model of the axis (in which measured endothelial, immune and neural variables forecast disease trajectory or treatment response in individual patients) does not yet exist. Graph theory, causal inference, and machine learning are enabling technologies that may eventually support such a model by identifying hub nodes and vulnerable network modules that recur across diseases. Their application to this axis, however, remains at the stage of method development. Constructing and prospectively validating a predictive model is therefore a principal unmet need. It will require longitudinal, multimodal datasets that are not yet available in most of the disease categories discussed here.

## 5 Clinical translation of immune-endothelial-targeted strategies

### 5.1 Biomarkers and diagnostics

Capturing the immune-endothelial-neural axis *in vivo* requires integration of fluid biomarkers, imaging, and vascular functional assessment, because no single marker can reflect its multidimensional changes.

#### 5.1.1 Peripheral blood markers

The most accessible indicators of endothelial activation include soluble adhesion molecules (sICAM-1, sVCAM-1, sE-selectin), vWF, and angiopoietin-2 (Ang-2). Elevated sICAM-1/sVCAM-1 reflects shedding from activated endothelial surfaces and correlates with recurrent stroke risk, white matter lesion burden, and cognitive decline rate in CSVD and VCID populations. vWF, released under endothelial injury and high shear stress, is an indirect marker of microthrombus formation. Ang-2, upregulated during basement membrane remodeling, associates with BBB breakdown and vascular fragility (Page and Liles, 2013; Gurnik et al., 2016).

Circulating inflammatory cytokines (TNF-α, IL-1β, IL-6, IL-17, IFN-γ) and their regulatory counterparts (IL-10, TGF-β) define the inflammatory background to which endothelial cells are exposed. Persistently elevated IL-6, for instance, correlates with white matter lesion volume in CSVD and VCID. Peripheral immune cell profiling by flow cytometry (Th1/Th17/Treg ratios, CCR2^+^ monocyte proportions, NLR, and monocyte-to-HDL ratio) estimates the activation state of potentially infiltrating immune subsets. Peripheral markers are attractive because they are readily collected and well-suited for repeated longitudinal assessment. Their main limitations, however, are poor spatial specificity and difficulty separating brain-derived endothelial changes from broader systemic endothelial alterations (Kadry et al., 2020).

#### 5.1.2 CSF markers

The CSF-to-serum albumin quotient (Q-alb), reflecting BBB leakage, is elevated in MS, CSVD, and early AD. CSF cytokines and chemokines (CXCL10, CCL2, IL-6, IL-8) indicate ongoing CNS immune activation, with CXCL10 and CCL2 levels correlating with T-cell and monocyte infiltration in MS and viral encephalitis. CSF vWF fragments, sICAM-1/sVCAM-1, and MMP-2/9 reflect direct microvascular endothelial injury. Postmortem immunohistochemistry of ICAM-1, VCAM-1, AQP4, and collagen IV in vessel walls provides gold-standard histopathological validation for fluid and imaging biomarkers (Zlokovic, 2008).

#### 5.1.3 Imaging and vascular functional assessment

MRI provides the richest structural information. SWI detects cerebral microbleeds, a fingerprint of microvascular fragility and repeated BBB microdisruption. FLAIR white matter hyperintensities reflect chronic endothelial dysfunction, hypoperfusion, and low-grade BBB leakage. DWI captures acute ischemic lesions; volumetric measures track chronic atrophy (González, 2012; Frisoni et al., 2010).

#### 5.1.4 Functional modalities assess the endothelial regulatory state in real time

CT and MR perfusion quantify CBF, CBV, and MTT, defining the ischemic penumbra in stroke and evaluating microcirculatory response to therapy. Arterial spin labeling (ASL) non-invasively captures subtle perfusion abnormalities in VCID and AD. CVR to CO_2_ inhalation provides a functional measure of NO signaling and neurovascular coupling integrity, and is therefore a sensitive marker of endothelial dysfunction. Extracranial measures, such as carotid intima-media thickness (IMT), pulse wave velocity (PWV), and retinal microvascular imaging, offer upstream views of how systemic vascular stress transmits to the cerebral microvasculature (Den Ruijter et al., 2012; van Sloten et al., 2015; Lemmens et al., 2020).

No individual modality is likely to be sufficient. Instead, serial multimodal assessment that incorporates peripheral biomarkers, CSF profiling, structural MRI, and vascular functional measures may provide a stronger framework for disease stratification, outcome prediction, and monitoring of therapeutic responses along the immune-endothelial-neural axis.

#### 5.1.5 Specificity and clinical validation

Several qualifications apply to the markers discussed above. First, exploratory research markers should be distinguished from markers with demonstrated clinical utility. For most of the molecules listed here, associations have been reported in single observational cohorts rather than established prospectively. Second, peripheral endothelial and inflammatory markers are not brain-specific. sICAM-1, sVCAM-1, E-selectin, vWF, Ang-2, and circulating cytokines are influenced by systemic inflammation, peripheral atherosclerotic burden, renal function, intercurrent infection, obesity, and medication. They therefore report the state of the systemic endothelium rather than of the cerebral microvasculature (Poggesi et al., 2016). Third, the imaging markers used in this review are likewise non-specific: white matter hyperintensities, lacunar infarcts and cerebral microbleeds can each arise from several vascular and non-vascular mechanisms. Cerebrovascular reactivity, moreover, reflects vascular function rather than endothelial phenotype specifically. Fourth, reported associations between circulating markers and white matter lesion burden or cognitive decline derive largely from single-cohort studies (Zhang et al., 2022; Shoamanesh et al., 2015). Effect sizes and thresholds are therefore not directly transferable between populations. Fifth, cerebrospinal fluid measures are more directly informative. The albumin quotient, CXCL10, CCL2, and MMP-9 have each been related to barrier leakage or to CNS immune activation (Puthenparampil et al., 2021; Kamat et al., 2012; Leppert et al., 2000). They require lumbar puncture, however, and are impractical for repeated monitoring. Sixth, although combinations of fluid, imaging, and functional markers are widely proposed for stratification, and are shown as such in Figure 3, they have not been prospectively validated as readouts of cerebral endothelial dysfunction: the multicenter validation programs established for this purpose have so far validated imaging markers such as cerebrovascular reactivity rather than blood-based panels (Wilcock et al., 2021; Liu et al., 2024). The multimodal approach is therefore presented here as a framework requiring prospective validation with prespecified thresholds and hard clinical endpoints.

**Figure 3:**
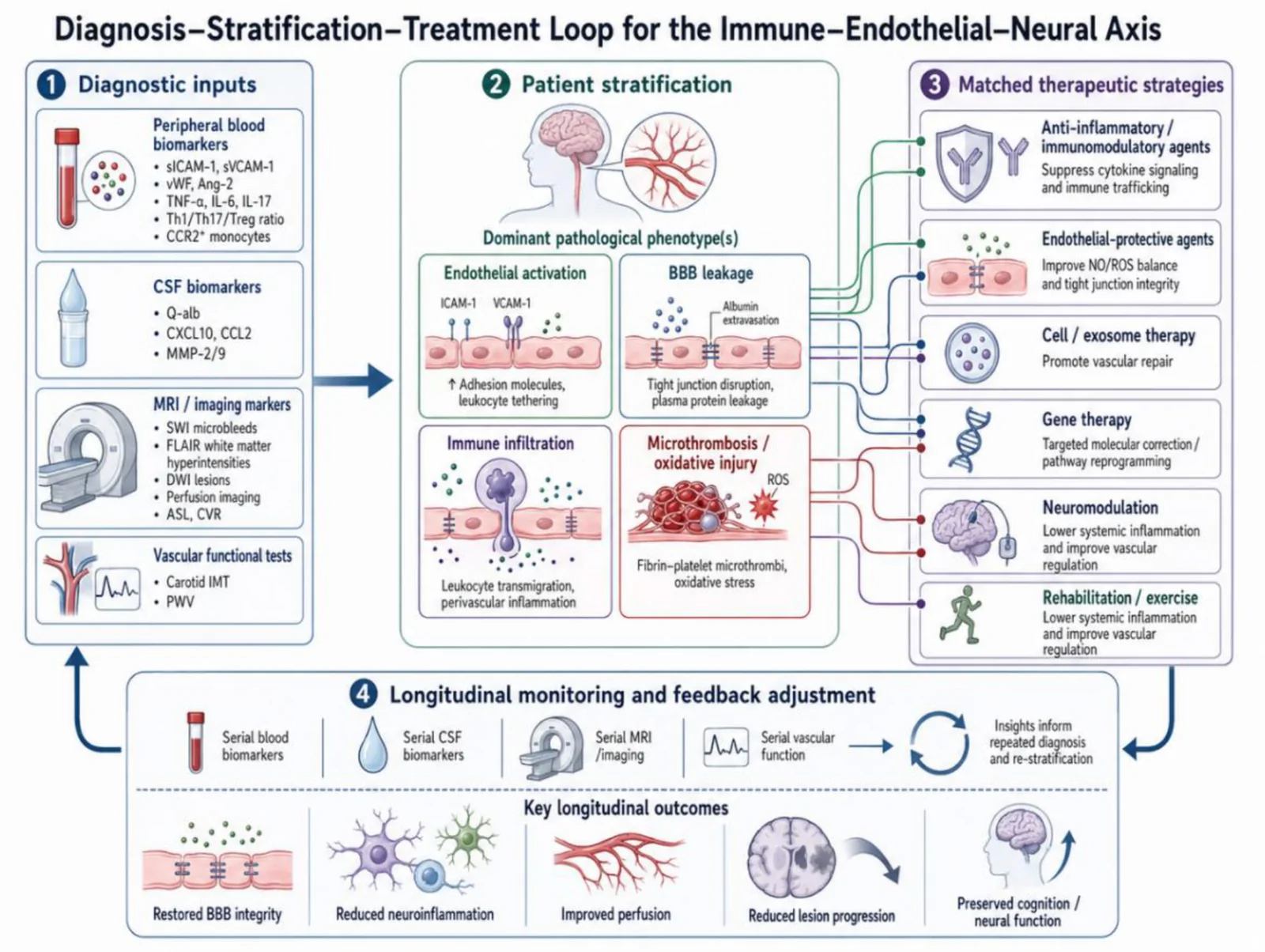
Diagnosis-stratification-treatment loop for the immune-endothelial-neural axis. Panel ① — Diagnostic inputs: Multimodal assessment combining peripheral blood biomarkers (sICAM-1, sVCAM-1, vWF, Ang-2, cytokines, immune cell ratios), CSF biomarkers (Q-alb, CXCL10, CCL2, MMP-2/9), MRI/imaging markers (SWI microbleeds, FLAIR white matter hyperintensities, DWI lesions, perfusion imaging, ASL, CVR), and vascular functional tests (carotid IMT, PWV). Panel ②–Patient stratification: Patients are classified by dominant pathological phenotype—endothelial activation (elevated adhesion molecules and leukocyte tethering), BBB leakage (tight junction disruption, plasma protein extravasation), immune infiltration (leukocyte transmigration, perivascular inflammation), or microthrombosis/oxidative injury (fibrin-platelet microthrombi, ROS). Panel ③–Matched therapeutic strategies: Six treatment modalities are linked to specific phenotypes—anti-inflammatory/immunomodulatory agents (targeting endothelial activation and immune infiltration), endothelial-protective agents (targeting BBB leakage via NO/ROS balance and junctional integrity), cell/exosome therapy (promoting vascular repair), gene therapy (targeted molecular correction), neuromodulation (reducing systemic inflammation and improving vascular regulation), and rehabilitation/exercise (long-term anti-inflammatory and vascular benefits). Panel ④–Longitudinal monitoring: Serial biomarker, imaging, and vascular function assessments inform repeated re-diagnosis and re-stratification, with treatment goals including restored BBB integrity, reduced neuroinflammation, improved perfusion, reduced lesion progression, and preserved cognition. Colored lines connect specific pathologies to their most relevant therapeutic strategies, emphasizing mechanism-matched, phenotype-directed intervention.

### 5.2 Therapeutic strategies

Viewed through the immune-endothelial-neural axis, therapy is no longer about single targets (antiplatelet, anti-inflammatory, or vasodilatory in isolation) but about multinodal regulation across the entire axis (Figure 3).

The therapies discussed below differ by orders of magnitude in the strength of evidence that they modify the axis. They are therefore grouped by evidence level rather than by mechanism.

#### 5.2.1 Established clinical benefit

S1P receptor modulators exemplify dual immunomodulatory-endothelial action: by trapping lymphocytes in lymphoid tissue while strengthening endothelial barrier function, they target both upstream inflammation and BBB stability. This mechanism has been validated in multiple sclerosis (Kappos et al., 2010; Brinkmann et al., 2010). Statins combine lipid lowering with pleiotropic endothelial effects (upregulating eNOS, suppressing CRP and adhesion molecules, and improving vascular compliance), which explains part of their established benefit in secondary stroke prevention (Oesterle et al., 2017; Amarenco et al., 2006). Reperfusion by thrombolysis or thrombectomy restores perfusion but imposes a second endothelial insult through oxidative stress and BBB disruption. Combining reperfusion with endothelial-protective agents is therefore an active research priority (Lochhead et al., 2024).

#### 5.2.2 Therapies under prospective clinical investigation

Anti-IL-6R antibodies reduce relapse rates in neuromyelitis optica spectrum disorder and limit IL-6-driven BBB leakage and edema, although long-term IL-6 suppression raises infection concerns (Traboulsee et al., 2020). IL-1 antagonism reduces recurrent cardiovascular events in patients with residual inflammatory risk, in part by suppressing endothelial activation, adhesion molecule expression, and MMP release (Ridker et al., 2017). Translation to neurological disease, however, remains at an early stage. TNF blockers require careful patient selection, since systemic experience has been associated with occasional demyelinating events consistent with disruption of CNS immune balance.

#### 5.2.3 Observational and epidemiological evidence

RAAS blockers reduce angiotensin II-induced oxidative stress, inflammatory gene expression, and vasoconstrictive bias. Epidemiological studies associate their use with slower white matter lesion progression in cerebral small vessel disease. These data are observational, and confirmation in trials with axis-relevant endpoints is required.

#### 5.2.4 Preclinical-stage approaches

Antioxidants, Nrf2 activators, and Rho/ROCK inhibitors target the NO/ROS balance and junctional integrity and reduce BBB permeability in preclinical models. VEGF/Ang-Tie2 modulation aims to suppress maladaptive VEGF signaling while enhancing Tie2/Ang-1-mediated endothelial-pericyte stabilization (Augustin et al., 2009). Mesenchymal stem cells and endothelial progenitor cells act mainly through paracrine mechanisms. They release VEGF, Ang-1, HGF, BDNF, and extracellular vesicles that downregulate adhesion molecules, inhibit apoptosis and pyroptosis, promote junctional reconstruction, and shift microglia toward reparative phenotypes (Davis et al., 2021; Yan et al., 2022). Preclinical studies report reduced BBB leakage, white matter injury and cognitive deficits across stroke, ICH, CSVD, and neuroimmune models, but clinical evaluation remains at an early stage. Gene therapy approaches target adhesion molecules and chemokines, inflammatory and oxidative pathways, and cell death or repair pathways. Targeted brain endothelial transduction via AAV or non-viral carriers, however, remains the principal bottleneck. Non-invasive neuromodulation and structured aerobic exercise act largely through the reverse pathway, reducing systemic inflammatory tone and improving endothelium-dependent vasodilation, with evidence currently limited to small physiological studies. Taken together, these agents exert broad but moderate effects and are best regarded as candidate long-term foundational therapy pending trials with axis-relevant endpoints.

### 5.3 Traditional Chinese medicine and the immune-endothelial-neural axis

Multicomponent herbal formulations have long been used in cerebrovascular disease and cognitive impairment in East Asia, and they are relevant here for one specific reason. They act on several of the processes discussed above at once, and therefore provide a test case for whether multitarget intervention on the axis is feasible. This section considers only findings in which endothelial or immune mechanisms were measured directly; traditional terminology is not treated as evidence for a modern mechanism.

#### 5.3.1 Experimental evidence for endothelial and immune effects

A limited number of studies have assessed formulations against endothelial or immune endpoints. In experimental sepsis-associated encephalopathy, a compound formula reduced brain endothelial pyroptosis through the NLRP3/caspase-1/GSDMD pathway, lowered circulating IL-6 and TNF-α, and increased VE-cadherin and occludin expression in brain tissue (Song et al., 2026). In ischemic stroke and carotid atherosclerosis, certain formulations have been associated with reductions in hs-CRP, IL-6 and TNF-α and with improvements in lipid profiles and oxidative stress parameters (Yin et al., 2025). Smaller studies have reported changes in vWF, sICAM-1 and flow-mediated dilation, and transcranial Doppler studies report increased cerebral blood flow velocity with slower intima-media thickening. In clinical series, compound formulations added to standard care have been associated with improvements in neurological deficit scores (NIHSS), functional independence (Barthel Index), and cognitive measures (MMSE, MoCA), with occasional reports of reduced recurrence (Yin et al., 2025; Zhang et al., 2025; Gao et al., 2021).

#### 5.3.2 Limitations

These findings must be interpreted with caution, and the limitations are substantial. Most mechanistic data derive from cell culture and animal experiments. Clinical studies are generally small, of short duration and inadequately blinded, and they rely on symptom scales rather than hard endpoints. Multicomponent formulas vary between manufacturers and batches in active constituent content, and problems of adulteration, pesticide residues and heavy metal contamination further undermine reproducibility. Most mechanistic work also examines single compounds and isolated pathways rather than the multicellular, multitarget behavior of a complete formula.

#### 5.3.3 Requirements for testing TCM-derived interventions within the axis framework

Three requirements follow. First, trials should be designed with prespecified axis-relevant endpoints, combining endothelial biomarkers (sICAM-1, Ang-2, albumin quotient), neuroinflammatory markers and BBB-related imaging (microbleed burden, white matter hyperintensity progression, cerebrovascular reactivity), rather than symptom scales alone. Second, the active constituents of each formula and their quantitative ranges should be defined, and multi-omics and network pharmacology used to construct component-target-pathway maps. Third, quality control should be established from cultivation to manufacture so that multicenter trials are reproducible. Until these conditions are met, TCM-derived interventions are best regarded as a candidate source of multi-target compounds requiring rigorous testing rather than as an established component of axis-directed therapy (Zhang et al., 2024; Cui et al., 2023).

## 6 Challenges and future directions

### 6.1 Bridging the translational gap

Rodent models differ fundamentally from humans in immune composition, cerebrovascular architecture, and NVU cellular organization. MCAO and EAE produce high-intensity, single-cause injuries that poorly replicate the chronic, low-grade, multimorbid pathology of most clinical patients (Sommer, 2017; Macrae, 2011). *In vitro* systems, even advanced organ-on-chip and vascularized organoid platforms, lack the full complexity of aging, metabolic comorbidity, and long-term immune-vascular interaction. Future progress requires bidirectional reverse translation: defining immune-endothelial phenotypes in human cohorts through multi-omics and imaging, then redesigning models to reproduce specific patient subtypes rather than idealized disease archetypes. Human iPSC-derived 3D co-culture systems and large-animal models incorporating age, sex, and comorbidity variables will be essential intermediaries.

### 6.2 From static snapshots to dynamic trajectories

Most current studies rely on single time-point, single-tissue analyses, which are useful for group comparisons but blind to how the axis reorganizes across disease onset, progression, and recovery. Longitudinal designs combining serial intravital imaging, repeated CSF/blood sampling, and multi-time-point spatial transcriptomics are needed to construct dynamic trajectories rather than static slices. A biomarker elevation at day 3 post-stroke may not carry the same meaning as the same elevation at month 6 in CSVD; resolving these temporal dynamics is a prerequisite for stage-appropriate intervention.

### 6.3 Precision phenotyping over diagnostic labels

Traditional disease categories (stroke, VCID, MS) are increasingly inadequate for guiding therapy along the axis. Classification by immune-endothelial phenotype (for example, acute endothelial disruption with hemorrhagic risk, chronic low-grade activation with BBB microleakage, or immune-mediated direct attack) offers a more therapeutically informative taxonomy. This taxonomy is most useful when integrated with biomarker panels and imaging signatures (Montaner et al., 2020). Achieving this will require future cohort studies and clinical trials to incorporate immune–endothelial phenotypes as design variables from the outset.

### 6.4 Technology integration

Spatial multi-omics, single-cell sequencing, radiomics, and high-resolution intravital imaging generate data of unprecedented dimensionality. Machine learning and graph neural networks can integrate cell-cell interaction networks with imaging features to construct multilevel maps spanning molecules, cells, microstructure, and whole-brain function. Identifying feature combinations most strongly associated with stroke recurrence, cognitive decline, or treatment response could yield simplified indices for clinical phenotyping (van Dam-Nolen et al., 2022). Realizing this potential requires multicenter cohorts with harmonized protocols, shared biobanks, and collaborative platforms spanning neurology, immunology, vascular biology, computational science, and clinical epidemiology.

## 7 Conclusion

Neurovascular diseases include ischemic stroke, intracerebral hemorrhage, cerebral small vessel disease, vascular cognitive impairment, multiple sclerosis, and Alzheimer's disease. They have traditionally been classified and treated by their dominant clinical presentation: vascular, immune-mediated, or neurodegenerative. This review has argued that, while these categories provide practical clinical utility, they may mask an underlying pathological basis shared across disorders. That basis is the immune-endothelial-neural axis, a dynamic bidirectional system in which the peripheral immune system, cerebrovascular endothelium, and neural networks continuously modulate one another to determine disease initiation, progression, and outcome.

Taken together, current research supports three principal observations. First, neuroinflammation and endothelial dysfunction are not parallel processes but components of a self-amplifying circuit. Immune dysregulation activates the endothelium, which disrupts the BBB, which admits further immune cells and mediators, which deepens neuroinflammation. Neural injury and CNS inflammation, in turn, reshape systemic immune status through neuro-endocrine pathways. Second, this circuit is progressive. Endothelial cells pass through a continuum from quiescence to priming to activation to structural injury, with each step amplifying the next and becoming harder to reverse. Third, the circuit operates across all major disease categories, differing not in its existence but in its tempo, vascular scale, regional distribution, and the relative weight of its three limbs. A fourth, perhaps most consequential observation is that this circuit is tractable. Interventions at multiple levels, including immunomodulation, endothelial protection, BBB stabilization, and neuroregulation, have shown at least partial efficacy across diseases. Emerging tools also promise more precise engagement with specific axis components.

Therapeutically, the axis framework suggests a hierarchical therapeutic model organized along its three limbs: upstream control of excessive inflammation and immune imbalance; intermediate protection and repair of the endothelium and BBB; and downstream promotion of neural network recovery and plasticity. In specific diseases and individual patients, the key to precision intervention lies in identifying where along the axis the dominant pathology concentrates and deploying a matching combination of shared and disease-specific strategies.

TCM, characterized by multicomponent, multitarget formulations, is in principle well suited to this systems-level, multipathway pathology, and increasing experimental evidence supports endothelial-protective and immunomodulatory effects of specific monomers and formulas. However, rigorous multicenter trials with axis-relevant endpoints and standardized quality control remain essential before TCM-derived strategies can achieve broad international credibility.

## Glossary

Aβ, Amyloid-β; AD, Alzheimer's disease; AECAs, Anti-endothelial cell antibodies; Ang-1/2, Angiopoietin-1/2; AQP4, Aquaporin-4; ARBs, Angiotensin II receptor blockers; ASL, Arterial spin labeling; BBB, Blood-brain barrier; BDNF, Brain-derived neurotrophic factor; CAA, Cerebral amyloid angiopathy; CBF, Cerebral blood flow; CBV, Cerebral blood volume; CCL, C-C motif chemokine ligand; CNS, Central nervous system; CO_2_, Carbon dioxide; COX, Cyclooxygenase; CCR, C-C chemokine receptor; CSF, Cerebrospinal fluid; CSVD, Cerebral small vessel disease; CXCL, C-X-C motif chemokine ligand; CXCR, C-X-C chemokine receptor; CVR, Cerebrovascular reactivity; DAMPs, Damage-associated molecular patterns; DWI, Diffusion-weighted imaging; EAE, Experimental autoimmune encephalomyelitis; EPCs, Endothelial progenitor cells; eNOS, Endothelial nitric oxide synthase; FasL, Fas ligand; FLAIR, Fluid-attenuated inversion recovery; FMD, Flow-mediated dilation; GPR81, G-protein-coupled receptor 81; GPX4, Glutathione peroxidase 4; GSDMD, Gasdermin D; HGF, Hepatocyte growth factor; ICAM-1, Intercellular adhesion molecule-1; ICH, Intracerebral hemorrhage; IFN-γ, Interferon-gamma; IL, Interleukin; IMT, Intima-media thickness; iPSC, Induced pluripotent stem cell; IRF, Interferon regulatory factor; LFA-1, Lymphocyte function-associated antigen-1; MAC, Membrane attack complex; MCAO, Middle cerebral artery occlusion; MHC, Major histocompatibility complex; MMPs, Matrix metalloproteinases; MPO, Myeloperoxidase; MS, Multiple sclerosis; MSCs, Mesenchymal stem cells; MTT, Mean transit time; NF-κB, Nuclear factor kappa-B; NLRP3, NOD-like receptor protein 3; NETs, Neutrophil extracellular traps; NLR, Neutrophil-to-lymphocyte ratio; NMOSD, Neuromyelitis optica spectrum disorder; NO, Nitric oxide; NOX, NADPH oxidase; NVU, Neurovascular unit; PAMPs, Pathogen-associated molecular patterns; PD, Parkinson's disease; P-gp, P-glycoprotein; PGI_2_, Prostacyclin; PWV, Pulse wave velocity; Q-alb, CSF-to-serum albumin quotient; RAAS, Renin-angiotensin-aldosterone system; ROS, Reactive oxygen species; RNS, Reactive nitrogen species; scRNA-seq, Single-cell RNA sequencing; S1P, Sphingosine-1-phosphate; SPMs, Specialized pro-resolving mediators; SWI, Susceptibility-weighted imaging; TCM, Traditional Chinese medicine; TEER, Transendothelial electrical resistance; TLR, Toll-like receptor; TNF-α, Tumor necrosis factor-alpha; tPA, Tissue-type plasminogen activator; TRAIL, TNF-related apoptosis-inducing ligand; Tregs, Regulatory T cells; VCAM-1, Vascular cell adhesion molecule-1; VCID, Vascular cognitive impairment and dementia; VEGF, Vascular endothelial growth factor; vWF, von Willebrand factor; ZO-1, Zonula occludens-1.
